# MicroRNA-3148 Modulates Allelic Expression of Toll-Like Receptor 7 Variant Associated with Systemic Lupus Erythematosus

**DOI:** 10.1371/journal.pgen.1003336

**Published:** 2013-02-28

**Authors:** Yun Deng, Jian Zhao, Daisuke Sakurai, Kenneth M. Kaufman, Jeffrey C. Edberg, Robert P. Kimberly, Diane L. Kamen, Gary S. Gilkeson, Chaim O. Jacob, R. Hal Scofield, Carl D. Langefeld, Jennifer A. Kelly, Rosalind Ramsey-Goldman, Michelle A. Petri, John D. Reveille, Luis M. Vilá, Graciela S. Alarcón, Timothy J. Vyse, Bernardo A. Pons-Estel, on behalf of the Argentine Collaborative Group, Barry I. Freedman, Patrick M. Gaffney, Kathy Moser Sivils, Judith A. James, Peter K. Gregersen, Juan-Manuel Anaya, Timothy B. Niewold, Joan T. Merrill, Lindsey A. Criswell, Anne M. Stevens, Susan A. Boackle, Rita M. Cantor, Weiling Chen, Jeniffer M. Grossman, Bevra H. Hahn, John B. Harley, Marta E. Alarcόn-Riquelme, on behalf of the BIOLUPUS and GENLES networks, Elizabeth E. Brown, Betty P. Tsao

**Affiliations:** 1 Division of Rheumatology, University of California Los Angeles, Los Angeles, California, United States of America; 2 Division of Rheumatology and The Center for Autoimmune Genomics and Etiology, Cincinnati Children's Hospital Medical Center, Cincinnati, Ohio, United States of America; 3 U.S. Department of Veterans Affairs Medical Center, Cincinnati, Ohio, United States of America; 4 Department of Medicine, University of Alabama at Birmingham, Birmingham, Alabama, United States of America; 5 Department of Medicine, Division of Rheumatology, Medical University of South Carolina, Charleston, South Carolina, United States of America; 6 Department of Medicine, Keck School of Medicine, University of Southern California, Los Angeles, California, United States of America; 7 Arthritis and Clinical Immunology Program, Oklahoma Medical Research Foundation, Oklahoma City, Oklahoma, United States of America; 8 Department of Medicine, University of Oklahoma Health Sciences Center, Oklahoma City, Oklahoma, United States of America; 9 U.S. Department of Veterans Affairs Medical Center, Oklahoma City, Oklahoma, United States of America; 10 Department of Biostatistical Sciences, Wake Forest University Health Sciences, Wake Forest, North Carolina, United States of America; 11 Division of Rheumatology, Northwestern University Feinberg School of Medicine, Chicago, Illinois, United States of America; 12 Department of Medicine, Johns Hopkins University School of Medicine, Baltimore, Maryland, United States of America; 13 Department of Internal Medicine, University of Texas Health Science Center at Houston, Houston, Texas, United States of America; 14 Department of Medicine, University of Puerto Rico Medical Sciences Campus, San Juan, Puerto Rico; 15 Divisions of Genetics and Molecular Medicine and Immunology, King's College London, London, United Kingdom; 16 Department of Medicine, Sanatorio Parque, Rosario, Argentina; 17 Department of Internal Medicine, Wake Forest School of Medicine, Winston-Salem, North Carolina, United States of America; 18 Robert S. Boas Center for Genomics and Human Genetics, Feinstein Institute for Medical Research, North Shore LIJ Health System, Manhasset, New York, United States of America; 19 Center for Autoimmune Diseases Research, Universidad del Rosario, Bogota, Colombia; 20 Division of Rheumatology and Department of Immunology, Mayo Clinic, Rochester, Minnesota, United States of America; 21 Clinical Pharmacology Program, Oklahoma Medical Research Foundation, Oklahoma City, Oklahoma, United States of America; 22 Rosalind Russell Medical Research Center for Arthritis, Department of Medicine, University of California San Francisco, San Francisco, California, United States of America; 23 Division of Rheumatology, Department of Pediatrics, University of Washington, Seattle, Washington, United States of America; 24 Center for Immunity and Immunotherapies, Seattle Children's Research Institute, Seattle, Washington, United States of America; 25 Division of Rheumatology, School of Medicine, University of Colorado Denver, Aurora, Colorado, United States of America; 26 Department of Human Genetics, University of California Los Angeles, Los Angeles, California, United States of America; 27 Centro de Genómica e Investigación Oncológica (GENYO), Pfizer–Universidad de Granada–Junta de Andalucia, Granada, Spain; 28 Department of Epidemiology, University of Alabama at Birmingham, Birmingham, Alabama, United States of America; University of Oxford, United Kingdom

## Abstract

We previously reported that the G allele of rs3853839 at 3′untranslated region (UTR) of Toll-like receptor 7 (*TLR7*) was associated with elevated transcript expression and increased risk for systemic lupus erythematosus (SLE) in 9,274 Eastern Asians [*P* = 6.5×10^−10^, odds ratio (OR) (95%CI) = 1.27 (1.17–1.36)]. Here, we conducted trans-ancestral fine-mapping in 13,339 subjects including European Americans, African Americans, and Amerindian/Hispanics and confirmed rs3853839 as the only variant within the *TLR7-TLR8* region exhibiting consistent and independent association with SLE (*P*_meta_ = 7.5×10^−11^, OR = 1.24 [1.18–1.34]). The risk G allele was associated with significantly increased levels of *TLR7* mRNA and protein in peripheral blood mononuclear cells (PBMCs) and elevated luciferase activity of reporter gene in transfected cells. *TLR7* 3′UTR sequence bearing the non-risk C allele of rs3853839 matches a predicted binding site of microRNA-3148 (miR-3148), suggesting that this microRNA may regulate *TLR7* expression. Indeed, miR-3148 levels were inversely correlated with *TLR7* transcript levels in PBMCs from SLE patients and controls (R^2^ = 0.255, *P* = 0.001). Overexpression of miR-3148 in HEK-293 cells led to significant dose-dependent decrease in luciferase activity for construct driven by *TLR7* 3′UTR segment bearing the C allele (*P* = 0.0003). Compared with the G-allele construct, the C-allele construct showed greater than two-fold reduction of luciferase activity in the presence of miR-3148. Reduced modulation by miR-3148 conferred slower degradation of the risk G-allele containing *TLR7* transcripts, resulting in elevated levels of gene products. These data establish rs3853839 of *TLR7* as a shared risk variant of SLE in 22,613 subjects of Asian, EA, AA, and Amerindian/Hispanic ancestries (*P_meta_* = 2.0×10^−19^, OR = 1.25 [1.20–1.32]), which confers allelic effect on transcript turnover via differential binding to the epigenetic factor miR-3148.

## Author Summary

Systemic lupus erythematosus (SLE) is a debilitating autoimmune disease contributed to by excessive innate immune activation involving toll-like receptors (TLRs, particularly TLR7/8/9) and type I interferon (IFN) signaling pathways. TLR7 responds against RNA–containing nuclear antigens and activates IFN-α pathway, playing a pivotal role in the development of SLE. While a genomic duplication of *Tlr7* promotes lupus-like disease in the Y-linked autoimmune accelerator (*Yaa*) murine model, the lack of common copy number variations at *TLR7* in humans led us to identify a functional single nucleotide polymorphism (SNP), rs3853839 at 3′ UTR of the *TLR7* gene, associated with SLE susceptibility in Eastern Asians. In this study, we fine-mapped the *TLR7-TLR8* region and confirmed rs3853839 exhibiting the strongest association with SLE in European Americans, African Americans, and Amerindian/Hispanics. Individuals carrying the risk G allele of rs3853839 exhibited increased *TLR7* expression at the both mRNA and protein level and decreased transcript degradation. MicroRNA-3148 (miR-3148) downregulated the expression of non-risk allele (C) containing transcripts preferentially, suggesting a likely mechanism for increased *TLR7* levels in risk-allele carriers. This trans-ancestral mapping provides evidence for the global association with SLE risk at rs3853839, which resides in a microRNA–gene regulatory site affecting *TLR7* expression.

## Introduction

Systemic lupus erythematosus (SLE [OMIM 152700]) is a complex and heterogeneous autoimmune disease with a strong genetic component that is modified by environmental exposures. Although the detailed etiopathogenesis of SLE remains unknown, excessive innate immune activation involving toll-like receptors (TLRs, particularly TLR7/8/9) and type I interferon (IFN) has been recognized as an important pathogenic mechanism in the disease [1]. Therapeutics targeting the TLR/IFN pathway are in development for the treatment of SLE, with ongoing clinical trials investigating monoclonal antibodies against IFN-α and inhibitors for TLR7/TLR9 (reviewed in [2]). Recent genome-wide association (GWA) and follow-up studies have revealed the association of a number of polymorphic variants in genes encoding components of the TLR/type I IFN pathway with susceptibility to SLE (reviewed in [3], [4]), providing insights at the molecular level to refine our understanding of this dysregulated pathway in the predisposition to SLE.

Our previous study identified a single nucleotide polymorphism (SNP), rs3853839, in the 3′ UTR of an X-linked gene *TLR7* to be associated with SLE in 4,334 cases and 4,940 controls of Eastern Asian descent [5], providing the first convincing evidence for the genetic contribution of *TLR7* to human SLE. Individuals carrying the risk G allele exhibited increased *TLR7* transcripts and a more robust IFN signature than non-risk C allele carriers [5]. In this study, by fine mapping the *TLR7-TLR8* region, we confirmed that the previously reported functional SNP rs3853839, located within a predicted binding site of miR-3148, was most likely responsible for observed association with SLE in three populations of non-Asian ancestry. We demonstrated a differential miR-3148 modulation explaining the effect of allelic variation at rs3853839 on *TLR7* expression.

## Results

### Confirmation of the association between rs3853839 and SLE susceptibility in European American, African American, and Hispanic ancestries

**Figure 1:**
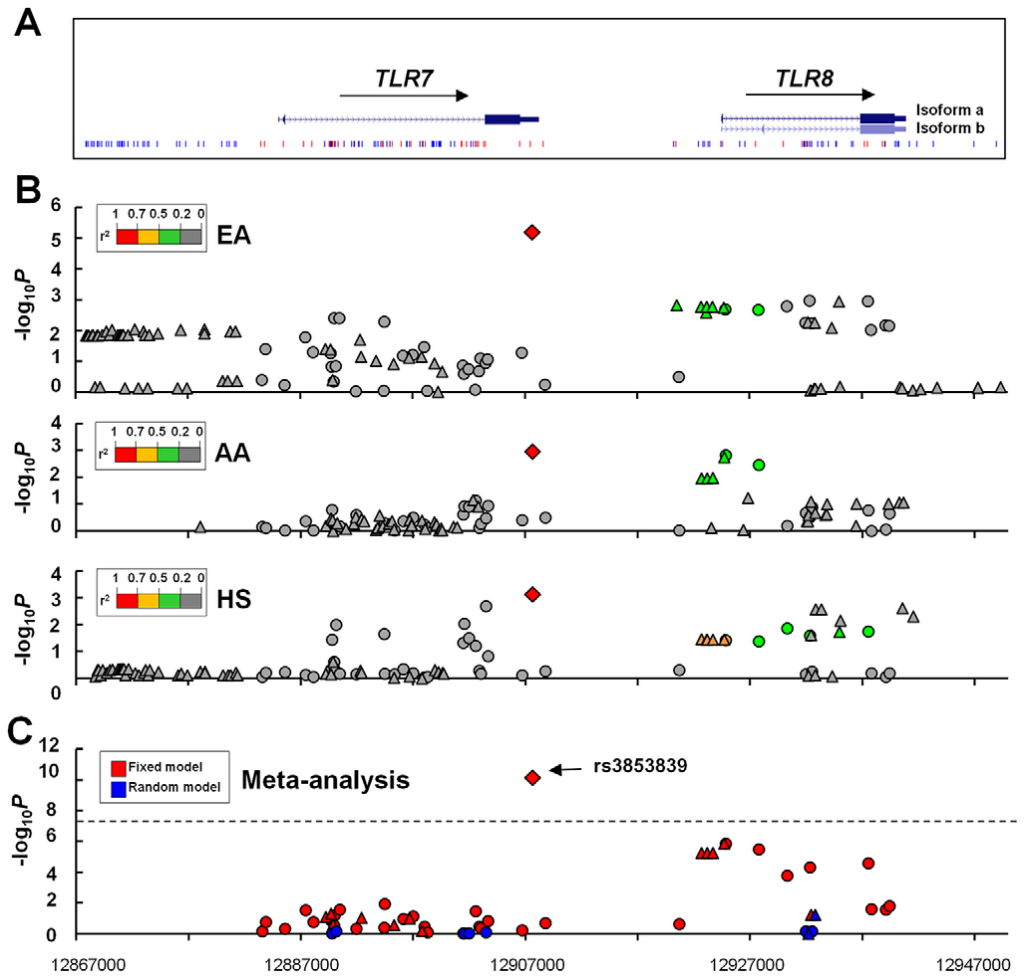
Allelic associations of SNPs in the *TLR7-TLR8* region with SLE. (A) The genomic structure of the *TLR7-TLR8* region and the location of all studied SNPs are indicated. (B) Association signals (−log_10_ *P*) are plotted against the position of each SNP (based on GRch37/hg19) in European Americans (EA), African Americans (AA), and Hispanics (HS). Genotyped and imputed SNPs are depicted with circles and triangles, respectively. The diamond identifies the *TLR7* 3′UTR SNP rs3853839. SNPs are highlighted using different colors according to their LD strength (r^2^) with rs3853839. (C) A trans-ancestral meta-analysis is conducted on 40 genotyped SNPs (circles) and 14 imputed SNPs (triangles) that are shared by the three ancestries (SNPs listed in Table S1) using fixed and random model, respectively. The dashed line represents the significance level of 5×10^−8^.

We conducted genotyping and imputation for genetic variants covering ∼80 kb of the *TLR7-TLR8* region on Xp22.2. After applying quality control measures, 41 genotyped SNPs and 57–75 imputed SNPs/INDELs (insertion-deletion) (varying among different ancestries) were assessed for association with SLE in unrelated cases and healthy controls of European American (EA, 3,936 cases vs. 3,491 controls), African American (AA, 1,679 vs. 1,934) and Hispanic enriched for the Amerindian-European admixture (HS, 1,492 vs. 807) descent (Figure 1A).

The strongest association signal was consistently detected at rs3853839 in the three ancestries, including EA (minor allele frequency of 20.3% in cases vs. 17.2% in controls, *P* = 6.5×10^−6^, OR [95%CI] = 1.23 [1.13–1.35]), AA (19.8% vs. 16.7%, *P* = 1.1×10^−3^, OR = 1.24 [1.09–1.41]) and HS (44.8% vs. 37.3%, *P* = 7.5×10^−4^, OR = 1.26 [1.10–1.43]) (Figure 1B, Table 1). After Bonferroni correction for multiple comparisons, the association of rs3853839 with SLE remained significant in EA and HS, and reached a nominal significance in AA. Combining the EA, AA and HS datasets, the meta-analysis *P* value of rs3853839 (*P_meta_* = 7.5×10^−11^, OR = 1.24 [1.18–1.34]) exceeded the commonly used threshold of 5×10^−8^ for genome-wide significance (Figure 1C, Table 1). Thus, the association of rs3853839 with SLE previously identified in Eastern Asians was confirmed in three non-Asian ancestries.

**Table 1:** Association of rs3853839 with SLE in multiple ancestries.

				Frequency				Trans-ethnic meta analysis	
Sex	Ethnicity	Case/Control	Test Allele	Case	Control	*P*	OR (95%CI)	*P_meta_*	OR (95%CI)
All	EA	3,936/3,491	G	20.3%	17.2%	6.5E-06	1.23 (1.13–1.35)		
	AA	1,679/1,934	G	19.8%	16.7%	1.1E-03	1.24 (1.09–1.41)		
	HS	1,492/807	G	44.8%	37.3%	7.5E-04	1.26 (1.10–1.43)		
	AS*	4,334/4,940	G	81.0%	77.0%	6.5E-10	1.27 (1.17–1.36)		
	Combined EA+AA+HS							7.5E-11	1.24 (1.18–1.34)
	Combined EA+AA+HS+AS							2.0E-19	1.25 (1.20–1.32)
Female	EA	3,592/2,340	G	20.3%	16.5%	1.9E-06	1.26 (1.15–1.39)		
	AA	1,543/1,342	G	19.7%	16.6%	3.4E-03	1.22 (1.07–1.40)		
	HS	1,365/727	G	44.9%	36.7%	2.5E-04	1.29 (1.13–1.48)		
	AS*	3,976/3,390	G	80.0%	77.0%	1.2E-07	1.24 (1.14–1.34)		
Male	EA	344/1,151	G	19.9%	20.0%	0.9	0.98 (0.73–1.33)		
	AA	136/592	G	22.8%	16.8%	0.1	1.46 (0.93–2.30)		
	HS	127/80	G	43.4%	49.4%	0.3	0.75 (0.42–1.34)		
	AS*	358/1,550	G	89.0%	77.0%	1.3E-06	2.33 (1.64–3.30)		

* AS: Previously published data in population of Eastern Asian descent (5).

Abbreviation: AS, Eastern Asian; AA, African American; EA, European American; HS, Amerindian/Hispanics; OR, odds ratio; CI, confidence interval.

Only six other SNPs within a relatively small interval of 5 kb spanning from *TLR7* 3′downstream to *TLR8* intron 1 were consistently associated with SLE (*P*<0.05) in EA, AA and HS (Table S1), and remained significant trans-ancestral meta-analysis *P* values after Bonferroni correction (5.5×10^−6^≤*P_meta_*≤1.3×10^−6^, Table S1). Linkage disequilibrium (LD) analysis revealed low LD strength between rs3853839 and these SNPs across non-Asian ancestries (r^2^<0.26, 0.37, and 0.51 in EA, AA and HS, respectively), but these 6 SNPs are in strong LD with each other and could be defined as a block (Figure S1). Among them, non-synonymous SNP rs3764880 (Met1Val) located at *TLR8* exon1 exhibited the strongest association (*P_meta_* = 1.3×10^−6^, OR = 1.15; Table S1). To distinguish whether the associations of these 6 SNPs with SLE were independent of rs3853839, we performed conditional haplotype-based association test. After conditioning on rs3853839, association signals detected at these 6 loci were completely eliminated in EA, AA and HS (Figure S1). In contrast, conditioning on rs3764880, a consistent association signal was detected at rs3853839 in EA and HS (Figure S1), indicating that the association signals detected at these 6 SNPs might be attributed to that of rs3853839.

Taken together, we confirmed rs3853839 as the only SNP in the *TLR7-TLR8* region showing an independent association with SLE across all three non-Asian ancestries. A meta-analysis by combining all datasets of Asian and non-Asian ancestries showed compelling evidence of association with SLE at rs3853839 (*P_meta_* = 2.0×10^−19^, OR = 1.25 [1.20–1.32], Table 1). Given the location of *TLR7* at X chromosome, we examined the allelic association of rs3853839 separately by gender. Of note, the sex-specific association of rs3853839 with SLE previously detected in Asian men [5] was not replicated in non-Asian ancestries (Table 1).

### Regulation of *TLR7* expression by rs3853839

**Figure 2:**
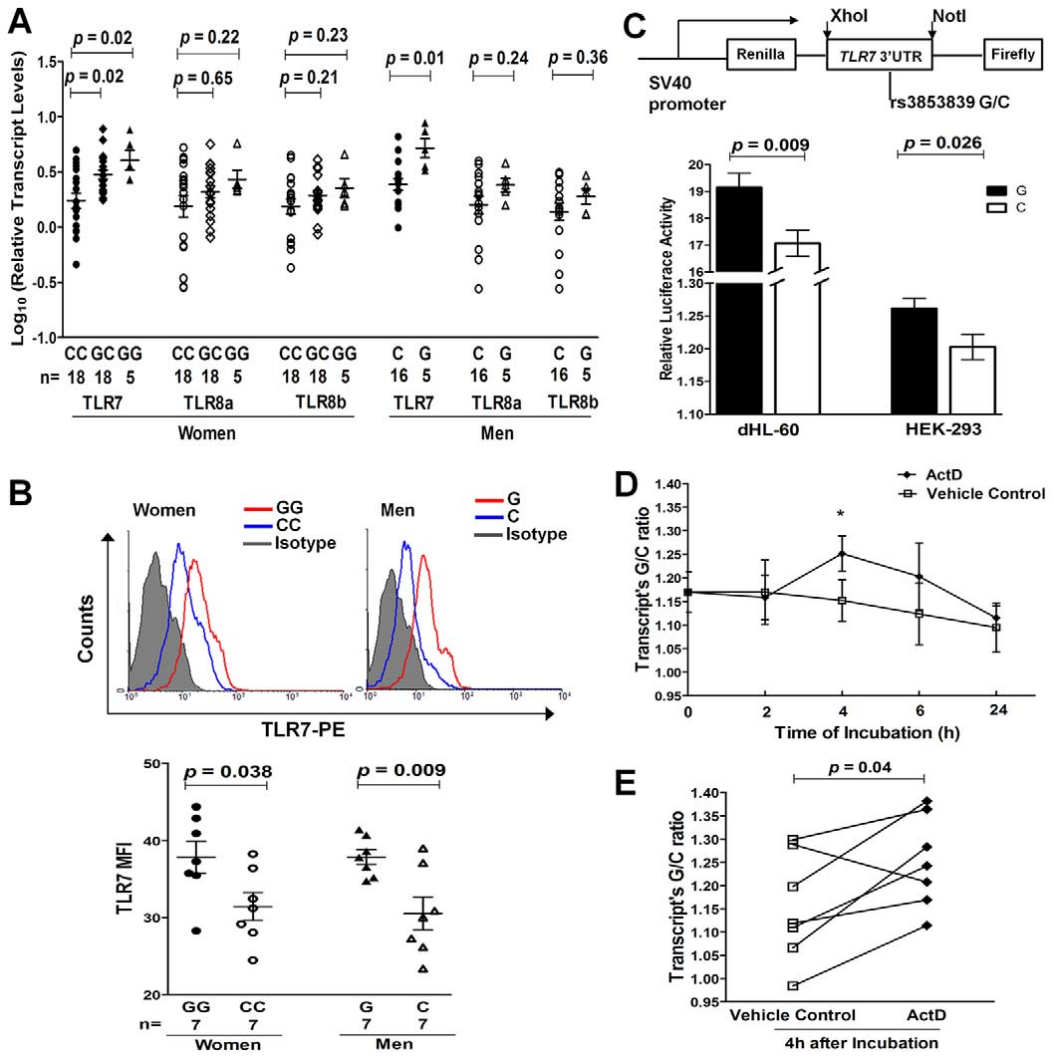
The SLE-risk G allele of rs3853839 confers elevated TLR7 expression through slower mRNA degradation. (A) Association of rs3853839 genotypes with *TLR7/8* transcript levels in EA normal PBMCs. Each symbol represents an individual and horizontal lines indicate mean ± SEM values. (B) Association of rs3853839 genotypes with TLR7 protein levels in normal PBMCs. FACS histograms show the log MFI values plotted against the cell counts for PBMCs in individuals carrying G or C allele, compared with isotype control. Results are from one representative pair (GG or G vs. CC or C) of 7 in each gender group. MFI of TLR7 expression in PBMCs is graphically depicted. Each symbol represents an individual and horizontal lines indicate mean ± SEM values. (C) Verification of the G allele conferring elevated expression of a luciferase reporter *in vitro*. *TLR7* 3′UTR segment bearing G or C allele of rs3853839 was cloned into the psiCHECK-2 reporter vector and luciferase activity was determined after 24 hours of transfection. Relative luciferase activity is Renilla/Firefly luciferase ratio. Data show the mean ± SEM and are representative of cumulative data from four independent experiments. (D) The kinetics of the G/C allele ratio in *TLR7* transcripts from PBMCs of healthy heterozygous individuals (n = 7) in the absence or presence of actinomycin D (ActD). The G/C allele ratio obtained in *TLR7* transcripts was normalized to that measured from gDNA of the same sample. Data are expressed as mean ± SEM at each time point and representative of cumulative data from two independent experiments with seven healthy donors. ^*^ *P*<0.05. (E) Summary of the G/C allele ratio in *TLR7* transcripts 4 hours after the addition of ActD or vehicle control (n = 7). Comparisons are between ActD and vehicle control cultures; *P* = 0.04; paired *t* test. FACS, Fluorescence-activated cell sorter; MFI, mean fluorescence intensity.

Given the convincing evidence for the trans-ancestral association of rs3853839 with SLE susceptibility, we then evaluated its effect on regulation of *TLR7/8* expression. Messenger RNA (mRNA) levels of *TLR7* and the two alternative *TLR8* isoforms were measured by real-time PCR in PBMCs from healthy EA individuals (n = 62). *TLR7* mRNA levels were significantly different among women (n = 41) carrying different genotypes of rs3853839 [*P* = 0.003, one-way analysis of variance (ANOVA)], in which the GG and GC carriers exhibited notably increased *TLR7* mRNA levels compared with the CC carriers [*P* = 0.02 for GG (n = 5) vs. CC (n = 18) and 0.02 for GC (n = 18) vs. CC, respectively, Student's *t* test; Figure 2A) and the number of rs3853839 risk G allele was significantly correlated with increased *TLR7* mRNA levels (R^2^ = 0.26, *P* = 8×10^−4^, linear regression test). Consistently, male G allele carriers (n = 5) also had significantly higher *TLR7* mRNA expression than male C allele carriers (n = 16) (*P* = 0.01, Figure 2A). There was no significant association of rs3853839 genotypes with mRNA levels of two *TLR8* isoforms in either women or men (Figure 2A). No sex differences in *TLR7* or *TLR8* mRNA levels were observed between individuals carrying the same genotype [GG women vs. G men: *P* = 0.41 (*TLR7*), 0.63 (*TLR8a*) and 0.50 (*TLR8b*); CC women vs. C men: *P* = 0.10 (*TLR7*), 0.91 (*TLR8a*) and 0.65 (*TLR8b*)]. These results were in accordance with our previous observations in Chinese [5], supporting the importance of rs3853839 in regulating *TLR7* rather than *TLR8* gene expression.

We assessed the intracellular expression of TLR7 and TLR8 proteins by flow cytometry in PBMCs from 7 pairs of healthy women (GG vs. CC) and men (G vs. C), respectively. Of the 7 pairs of individuals in each gender, 4 pairs were of EA descent and 3 pairs were Asians. Compared with C allele carriers, G allele carriers had significantly higher TLR7 protein levels in PBMCs (*P* = 0.038 and 0.009 in women and men, respectively; Figure 2B), especially in CD19^+^ B cells and CD14^+^ monocytes (Figure S2). No significant association between rs3853839 genotypes and TLR8 protein levels was observed in either total PBMCs or in specific cell subsets (Figure S3).

We next performed luciferase reporter assays to further confirm the functional effect of rs3853839 on *TLR7* expression. PCR-amplified *TLR7* 3′UTR fragments with either the G or C allele of rs3853839 were cloned downstream of an SV40 promoter-driven *Renilla* luciferase gene in the psiCHECK-2 vector, which also contained a firefly luciferase gene to serve as an internal transfection normalization control (Figure 2C). Constructs were then transiently transfected into either HEK-293 or differentiated HL-60 (dHL-60, neutrophil-like cells) cells. After 24 hours, cell lysates transfected with the G-allele construct showed significantly higher luciferase activity than those transfected with the C-allele construct in both HEK-293 and dHL-60 cells (*P* = 0.026 and 0.009, respectively; Figure 2C). Taken together, consistent results from *ex vivo* and *in vitro* studies indicated that the SLE-risk G allele of rs3853839 conferred elevated *TLR7* expression at the both mRNA and protein level.

### Allelic differences of rs3853839 in *TLR7* mRNA degradation rate

To explore the mechanism of rs3853839 in regulating *TLR7* mRNA turnover, we assessed allelic difference in *TLR7* mRNA degradation by pyrosequencing. We first determined the rs3853839 G/C allele ratio in genomic DNA (gDNA) and cDNA from healthy EA women (n = 7) carrying the GC genotype. The mean G/C allele ratio in cDNA was significantly higher than the theoretical ratio of 1 as detected in gDNAs (*P* = 0.02, Figure S4), indicating a higher expression of the G- than the C-allele containing *TLR7* transcripts in heterozygous PBMCs. The allelic specific expression analysis in EA was similar to our previous findings in Chinese [5], and confirmed the result of real-time PCR that the G allele of rs3853839 is associated with increased *TLR7* mRNA expression. Then, PBMCs were cultured in the absence or presence of the transcriptional inhibitor actinomycin D (ActD), and the G/C allele ratio in cDNA (normalized to that measured in gDNA) was determined after 0, 2, 4, 6, and 24 hours, respectively. As shown in Figure 2D and 2E, the G/C ratio in cDNA appeared to change over time when PBMCs were incubated with ActD and exhibited a statistical difference at the 4 hour point (*P* = 0.04), implicating slower degradation of the G allele- than the C allele-containing *TLR7* transcript in heterozygous PBMCs. The inhibitory effect of ActD on RNA synthesis was corroborated by a decrease in total *TLR7* mRNA level at increasing time points after the addition of ActD in PBMC aliquots measured by real-time PCR (Figure S5).

### Alteration of microRNA–3148–mediated modulation of *TLR7* expression by rs3853839

**Figure 3:**
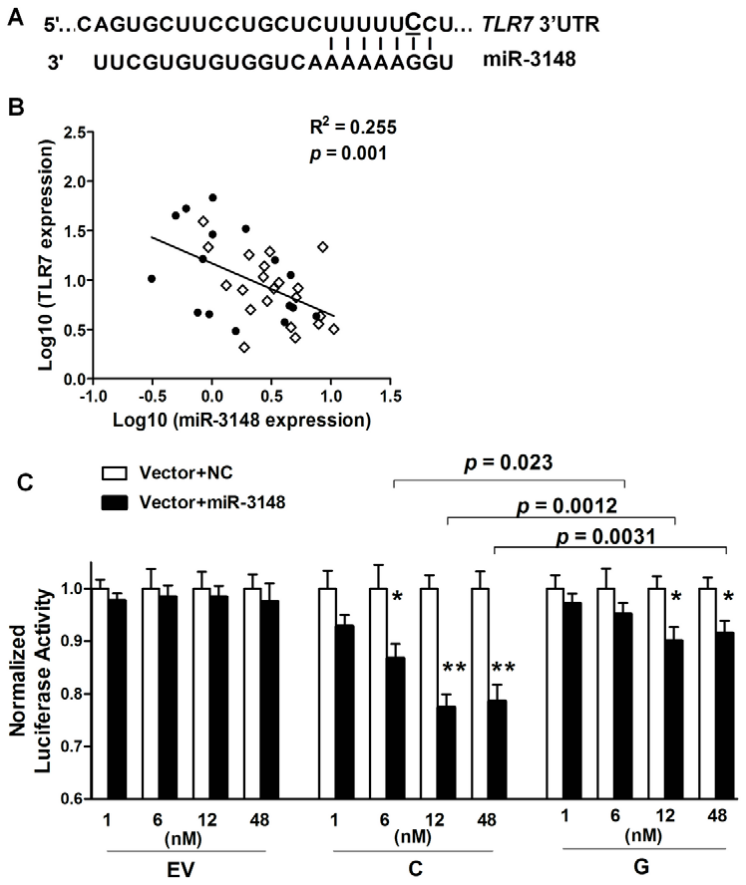
The SLE-risk G allele of rs3853839 displays reduced transcript modulation by miR-3148. (A) TargetScan's predicted miR-3148-binding site in *TLR7* 3′UTR. The C allele, rather than G allele of rs3853839 corresponds to the second base of this seed region. (B) Inverse correlation of miR-3148 and *TLR7* transcript levels in PBMCs from 16 patients with SLE (solid circles) and 21 controls (open diamonds). (C) HEK-293 cells were cotransfected with empty reporter vector (EV), luciferase constructs driven by *TLR7* 3′UTR segment containing either C or G allele of rs3853839 and increasing concentrations (1, 6, 12, and 48 nM) of miR-3148 or nontarget control (NC) mimics. Luciferase activity was determined 24 hours after transfection. Normalized luciferase activity is the Renilla/Firefly ratio of miR-3148-treated reporter vector compared with the same NC-treated reporter vector. Data show the mean ± SEM and are representative of cumulative data from three independent experiments. *P* = 0.0003 over all miR-3148-treated C-allele vector groups, and not significant over all miR-3148-treated G-allele or empty vector groups (ANOVA test). P* = 0.02, P**<0.0001 (Student's *t* test) for the comparison of indicated groups.

MicroRNAs (miRNAs) that bind to target sequences located within the 3′UTR of mRNAs by base pairing have been shown to result in accelerated mRNA turnover or translation repression [6]. Single nucleotide change either within or around the sequence of miRNA target sites can potentially alter the base-pairing patterns and affect miRNA-mediated regulation [7], [8]. The updated TargetScan database (Release 6.2; http://www.targetscan.org) indicates that rs3853839 is located within a binding site of miR-3148, where the non-risk allele (C), but not the risk allele (G), is predicted to match miR-3148 at the second position (Figure 3A). We hypothesized that the C to G variation of rs3853839 could reduce the binding and regulation incurred by miR-3148, therefore, leading to dysregulated *TLR7* expression. We first showed that transcript levels of miR-3148 and *TLR7* were inversely correlated in PBMCs from 16 patients with SLE and 21 healthy controls (R^2^ = 0.255, *P* = 0.001; Figure 3B), suggesting the possible regulation of *TLR7* expression by miR-3148. Next, to verify whether allelic variation of rs3853839 affects the interaction of miR-3148 with *TLR7* 3′UTR, psiCHECK-2 vectors containing *TLR7* 3′UTR segment with either the C or G allele of rs3853839 were cotransfected with various doses of miR-3148 or nontarget control mimic into HEK-293 cells. As shown in Figure 3C, we observed significant dose-dependent miR-3148-mediated decrease in luciferase activity for the C-allele construct (*P* = 0.0003 over all miR-3148-treated C-allele vector groups, ANOVA test), but not for the G-allele construct (*P* = 0.14). Cotransfection with miR-3148 at a concentration of 6, 12, and 48 nM, respectively, led to greater than two-fold reduction of luciferase activity in the C-allele than the G-allele construct [reduction in C-allele vs. G-allele construct: 13.2% vs. 4.8%, *P* = 0.023 (6 nM); 22.5% vs. 9.9%, *P* = 0.0012 (12 nM); 21.4% vs. 8.5%, *P* = 0.0031 (48 nM)]. These data supported the bioinformatic prediction that miR-3148 directly targets *TLR7* 3′UTR and the C to G variation of rs3853839 within the binding site alters the inhibitory effect of miR-3148 on modulating *TLR7* expression.

## Discussion

Fine-mapping of the *TLR7-TLR8* region with high-density genetic markers based on large scale genotyping and imputation confirmed SNP rs3853839 at *TLR7* 3′UTR as the most likely causal variant responsible for the association of *TLR7-TLR8* region with SLE in populations of EA, AA and HS ancestry. In accordance with our previous observation in Asians [5], we detected elevated *TLR7* expression at both mRNA and protein levels in PBMCs from EA homozygous risk G allele carriers, as well as a higher level of the risk than the non-risk allele-containing *TLR7* transcripts in EA heterozygous PBMCs. The fact that two distinct ancestries share the same genotype-phenotype association implicates an important regulatory effect of rs3853839 on *TLR7* expression. Toward this end, we have extended functional studies showing slower degradation of the risk allele-containing *TLR7* transcripts in heterozygous PBMCs and regulation of *TLR7* expression by miRNA-3148 that targets 3′UTR at the position of rs3853839. Finally, we showed that the presence of the risk G allele resulted in reduced suppression by miRNA-3148, suggesting a likely mechanism for increased *TLR7* expression in risk-allele carriers.

The importance of TLR7 upregulation on mediating autoimmune responses has been addressed in murine models of SLE. The Y-linked autoimmune accelerator (*Yaa*) modifier, suggested mainly due to *Tlr7* gene duplication, provides a prime example of TLR7 dysregulation leading to autoreactivity and inflammatory pathology [9]–[11]. Increasing *Tlr7* gene dosage via generation of transgenic mice results in development of systemic autoimmunity, the severity of which directly correlates with the degree of *Tlr7* overexpression [12]. Increased *Tlr7* gene dosage promotes autoreactive lymphocytes activation, dendritic cells proliferation, and secretion of proinflammatory cytokines and IFN-α [12], which in turn upregulates TLR7 expression, leading to a feedback loop exacerbating autoimmunity [13]. In patients affected with SLE, up-regulated expression of *TLR7* mRNA has been reported in PBMCs and B cells [14], [15]. Although a copy number variation (CNV) study in Mexican population showed increased *TLR7* copies in childhood-onset SLE patients [16], no evidence for common CNVs at the *TLR7-TLR8* region has been identified in individuals of diverse ancestries through our previous study by three independent methods including quantitative real-time PCR, PmeI pulsed-field gel electrophoresis and Southern blot [5], two recent studies using customized CGH platforms [17], [18] as well as other studies listed in the Database of Genomic Variants (http://projects.tcag.ca/variation; the latest version released in November 2010), suggesting that mutations similar to Yaa are not a frequent feature of human SLE. The current study identifying genetic variations conferred by a regulatory SNP in *TLR7* expression and SLE susceptibility suggests that murine models provide profound clues to human genetics if we look beyond the specific mutations identified in the relevant pathways.

Unlike our findings in Asians that both sexes showed association [5], the impact of rs3853839 on risk for SLE was only observed for women in the non-Asian datasets (Table 1). Given the low prevalence of SLE in men, it is often challenging to collect a large enough number of affected men in a given population. Under the assumption that the associated G allele confers genetic risk with an odds ratio of 1.26 in EA, 1.22 in AA and 1.29 in HS subjects (ORs were determined in female datasets), and considering *P*<0.05 as the threshold of significance, the power estimate for female samples in each ancestry reaches more than 85%, whereas for male samples it is only 50% in EA, 19% in AA and 25% in HS dataset. Thus, there was clearly inadequate power to evaluate this association in AA and HS men. Despite a relatively robust sample size of EA men (344 SLE vs. 1,151 controls), a significantly higher G allele frequency was observed in male than female controls (20.0% vs. 16.5%, *P* = 0.005), contributing to the difficulty in assessing association with SLE in EA male subjects.

To our knowledge, the association of rs3853839 (or its tag SNP) with SLE has not been reported in four SLE GWA studies in European-derived populations [19]–[22] and three GWA studies in Asians [23]–[25]. According to the 1000 Genomes Project data, rs3853839 locates in a region with poor LD structure and cannot be tagged by any known SNP at the *TLR7-TLR8* region with r^2^>0.65. The SNP rs850632, located at *TLR7* 3′downstream, shows the strongest LD with rs3853839 in Europeans (r^2^ = 0.38) and Asians (r^2^ = 0.65). However, neither rs3853839 nor rs850632 has been included in predesigned commercial genotyping arrays of those GWA studies, resulting in the absence of associations. Even if rs3853839 was genotyped, the published GWA studies might have inadequate statistical power to capture its association in the initial discovery analyses [5].

Evidence of other *TLR7* polymorphisms associated with SLE has been reported, including two intronic SNPs (rs179019 and rs179010) found in Japanese population [26] and an exonic SNP (rs179008) in individuals from Southern Brazil [27]. The reported associations were modest due to limited sample size of these studies (less than 400 cases and 450 controls), and none of them have been confirmed by the current fine-mapping study using a large collection of EA, AA and HS cases-controls (Table S1). *TLR8* polymorphisms have been described in infectious diseases [28], [29] with a genetic effect localized to a functional variant at exon 1 (rs3764880, Met1Val). The G allele of rs3764880, which abolishes a putative start codon within the alternative *TLR8* transcript isoform a (Figure 1A), conferred a protective effect on susceptibility to pulmonary tuberculosis in Indonesian and Russian men [28], as well as on HIV disease progression in Germans [29]. Our data showed a significantly increased frequency of rs3764880-G allele in SLE than healthy controls in the three non-Asian datasets; however, its association with SLE was dependent on that of *TLR7* SNP rs3853839. Other variants at the *TLR7*-*TLR8* region showed either weaker association than rs3853839 in trans-ancestral meta-analysis or association uniquely in EA or HS. Taken together, these data support rs3853839 as the most likely polymorphism associated with SLE shared by multiple ancestries. Although imputation facilitated our ability to capture common variants (MAF>1%), further refinement in genetic effects of rare variants (MAF<1%) is needed by deep sequencing of this locus, especially the intergenic region between *TLR7* and *TLR8* that was not well imputed in this study.

Variations in 3′UTR regions may be important in gene regulation. To date, expression quantitative trait loci (eQTL) mapping has been widely used for characterization of SNPs that affect gene expression [30]. Although the *TLR7* expression has been measured in previous whole-genome eQTL studies, currently only those using EBV-transformed lymphoblastoid cell lines of 1000 Genomes Project individuals provide publically available genotyping data of rs3853839. Based on the study by Stranger et al [31], we found that CG carriers of rs3853839 showed elevated *TLR7* expression compared with CC carriers in YRI women (*P* = 0.012). In male individuals, the G allele of rs3853839 showed a trend of association with elevated *TLR7* expression in CHB+JPT, CEU and YRI men, and the association was significant when combining all male data (*P* = 0.014). These findings are consistent with our results that rs3853839 alleles are associated with differential *TLR7* expression.

An important finding of this study is that the SLE-associated variant rs3853839 confers a genetic effect on modulation of *TLR7* expression by an epigenetic factor miR-3148. Accumulating evidence suggests that miRNAs are fine tuners of TLR signaling pathways [32]. Regulation by miRNA may occur at various levels of TLR pathways by targeting adaptor molecules, downstream regulators and cytokines (reviewed in [32], [33]). However, few studies point to TLR themselves (e.g. TLR2 and TLR4) being directly targeted by miRNAs [34], [35]. Using algorithms from TargetScan, only the newly identified human miR-3148 [36], which is not evolutionarily conserved among mammals, is predicted to bind *TLR7* 3′UTR sequences at the position of rs3853839. The inverse correlation of miR-3148 and *TLR7* levels in PBMCs, along with functional validation by reporter gene assay, confirms an inhibitory effect of miR-3148 on regulating *TLR7* expression and allelic variation of rs3853839 affecting miRNA-mRNA interactions. Further study will focus on investigating miR-3148 expression patterns in specific immune cell types, assessing biological impacts of changes in miR3148-mediated TLR7 expression on downstream immune responses, and evaluating roles of other miRNAs that target sequences in the vicinity of rs3853839. Of interest, an unconventional role for miRNAs has been identified as endogenous activators for RNA-sensing receptors (TLR7/8) in a cell- or tissue-type specific manner [37], [38]. Therefore, miRNA regulation in TLR7 signaling is more complicated than we expected and further functional studies showing the exact effects of miRNAs on TLR7 responses are warranted.

In summary, we have advanced our previous study by showing rs3853839 (at *TLR7* 3′UTR) as the most likely polymorphism responsible for the association of *TLR7-TLR8* region with SLE in individuals of EA, AA and HS ancestry, and have characterized a differential miR-3148 modulation which explains the effect of allelic variation of rs3853839 on *TLR7* expression. Our study highlights the importance of *TLR7* as a shared genetic contributor to SLE in multiple ancestries, and provides evidence that microRNA acts as a negative regulator to control *TLR7* expression, suggesting the possibility of miRNA-based therapies for amelioration of autoimmune diseases such as SLE where excessive TLR7 activation exists.

## Materials and Methods

### Ethics statement

Written informed consent was obtained from all study participants and each participating institution had Institutional Review Board (IRB) approval to recruit samples. The overall study was approved by the IRB of the Oklahoma Medical Research Foundation (OMRF).

### Subjects

To test the association of *TLR7-TLR8* with SLE, we used a large collection of case-control subjects from the collaborative Large Lupus Association Study 2 (LLAS2), including European American (4,248 cases vs. 3,818 controls), African American (1,724 cases vs. 2,024 controls), and Hispanic enriched for the Amerindian-European admixture (1,622 cases vs. 887 controls). African Americans included 286 Gullahs (155 cases vs. 131 controls), who are subjects with African ancestry. Cases were defined by meeting at least four of the 1997 American College of Rheumatology (ACR) revised criteria for the classification of SLE [39].

### SNP genotyping and quality control

DNA samples were processed at the Lupus Genetics Studies Unit of OMRF. SNP genotyping was performed using an Illumina custom bead array on the iSCAN instrument for 47 SNPs covering the *TLR7-TLR8* region on Xp22.2 and 347 admixture informative markers (AIMs). SNPs meeting the following criteria were included in the association analysis: well-defined cluster scatter plots, SNP call rate >90%, minor allele frequency >1%, total proportion missing <5%, *P*>0.05 for differential missing rate between cases and controls, and Hardy-Weinberg proportion (HWP) test with a *P*>0.01 in controls and *P*>0.0001 in cases.

Subjects with genotype missing rate >10% (due to low quality), shared identical by descent >0.4 or showing mismatch between the reported and estimated gender were removed. The global ancestry of each subject was estimated based on genotype of AIMs using principal components analysis [40] and ADMIXMAP [41], as described in another LLAS2 study [42], and then genetic outliers were removed.

Finally, a total of 13,339 unrelated subjects, including European Americans (EA: 3,936 cases vs. 3,491 controls), African Americans (AA: 1,679 vs. 1,934; composed of 92.5% of African Americans and 7.5% Gullahs) and Hispanics enriched for the Amerindian-European admixture (HS: 1,492 vs. 807), were analyzed for 41 genotyped SNPs of *TLR7-TLR8*.

### Imputation methods

Imputation was performed at 12.86–12.95 Mb on Xp22.2 using IMPUTE 2.1.2 [43], with SNP/INDEL genotypes of 381 Europeans, 246 Africans and 181 Americans from the 1000 Genomes Project (“version 3” of the Phase 1 integrated data, March 2012 release) as references in imputation for our EA, AA and HS subjects, respectively. Imputed genotypes had to meet information score of >0.9, as well as the quality control criteria as described above. After imputation, we obtained an additional 75 variants for EA, 57 for AA and 63 for HS (the number varied based on LD structure) for further analysis.

### Real-time PCR

Total RNA was purified with TRIzol reagent (Invitrogen) from PBMCs and reverse-transcribed into cDNA with Superscript II Reverse Transcription kit (Invitrogen). The mRNA levels of *TLR7* (NM016562.3) and *TLR8* (isoform a: NM138636.4 and isoform b: AF246971.1) were measured by quantitative real-time PCR using TaqMan assays (*TLR7* probe: Hs00152971_m1; *TLR8 isoform a* probe: Hs00607866_mH; *TLR8 isoform b* probe: Hs00152972_ml, Applied Biosystems). All samples were run in triplicate. Relative expression levels of *TLR7* and *TLR8* were normalized to the level of *RPLP0*, calculated by the 2^−ΔΔCt^ method and Log10 transformed. The association of rs3853839 with mRNA levels of *TLR7* or *TLR8* was evaluated using ANOVA, Student's *t* and linear regression test.

To examine the correlation of miR-3148 and *TLR7* mRNA levels, total RNA enriched in small RNAs were isolated from PBMCs using mirVanaTM miRNA isolation kit (Invitrogen), followed by reverse transcription with TaqMan MicroRNA Reverse Transcription kit (Applied Biosystems; for detecting miR-3148) and Superscript II Reverse Transcription kit (Invitrogen; for detecting *TLR7*), respectively. The miR-3148 level was quantified using Taqman MicroRNA Expression assay (Applied Biosystems), and the *TLR7* level was measured using the same probe as described above. All samples were run in triplicate. Relative expression levels of miR-3148 and *TLR7* were normalized to the level of snRNA U6 and *RPLP0*, respectively, calculated by the 2^−ΔΔCt^ method and Log10 transformed. Association between transcript levels of *TLR7* and miR-3148 was evaluated using linear regression test.

### Flow cytometry

Four-color flow cytometry was performed to investigate intracellular expression of TLR7 and TLR8 in PBMCs from healthy EA and Asian individuals who were homozygous for rs3853839 (7 pairs of G-allele vs. C-allele carriers in each gender group). Freshly isolated PBMCs were incubated with 2% pooled human serum to block nonspecific binding to Fcγ receptors and then incubated with peridinin chlorophyll protein (PerCP)-conjugated anti-human CD3, allophycocyanin (APC)-conjugated anti-human CD19 and phycoerythrin (PE)-conjugated or fluorescein isothiocyanate (FITC)-conjugated anti-human CD14 (Miltenyi Biotec) to identify T cell, B cell and monocyte subpopulations, respectively. For intracellular staining, PBMCs were fixed in Fixation buffer (R&D Systems) for 10 minutes at room temperature, washed twice in Permeabilization/Wash buffer (R&D Systems) and stained with PE-conjugated mouse anti-human TLR7 mAb (R&D Systems) and FITC-conjugated mouse anti-human TLR8 mAb (Imgenex) for 1 hour at room temperature. Background fluorescence was assessed using appropriate isotype- and fluorochrome-matched control antibodies. Cells were collected and analyzed by FACSCalibur flow cytometer equipped with the manufacturer's software (CellQuest; BD Biosciences). Student's *t* test was used to compare protein levels of TLR7 or TLR8 in PBMCs from individuals of different genotypes.

### Plasmid construction and luciferase reporter assay

The fragment of *TLR7* 3′-UTR bearing the G or C allele of rs3853839 was amplified by PCR from genomic DNA of subjects homozygous for the G or C allele using the following primers: 5′-TGTCTCGAGCCCTTCTTTGCAAAAC-3′ (forward) and 5′-AGAGCGGCCGCTAGTTGGCTCCAGCAAT-3′ (reverse). The PCR products were inserted into the downstream of the *Renilla* luciferase gene in the reporter vector psiCHECK-2 (Promega) by digestion using the restriction enzymes *Not* I and *Xho* I. The psiCHECK-2 vector also contained a firefly luciferase gene to serve as an internal transfection normalization control. All constructs were sequenced to assure proper orientation and authenticity in the vector.

HEK-293 (human embryonic kidney cell line) and HL-60 (human leukemic cell line) cells were obtained from the American Type Culture Collection (ATCC). HEK-293 cells were maintained in Dulbecco's modified Eagle's medium supplemented with 10% FBS, seeded on a 24-well plate at a concentration of 2×10^5^ cells/well, and transiently transfected using Lipofectamine 2000 (Invitrogen) with 1 µg of either rs3853839 G or C reporter construct. HL-60 cells are predominantly a neutrophilic promyelocyte (precursor) and can be induced to differentiate to neutrophil-like cells when grown in RPMI 1640 medium with 15% FBS plus 2 mM L-glutamine, 25 mM HEPES and 1.25% DMSO [44]. Differentiated HL-60 cells seeded on 24-well plates (2×10^6^ cells/well) were electroporated with 3 µg of report construct on a nucleofector device (Amaxa). The luciferase activity in total cell lysates was measured after 24 hours using a dual luciferase reporter assay system (Promega). *Renilla* luciferase activities were normalized to firefly luciferase activities. Each transfection was performed in quadruplicates and triplicates for HEK-293 and HL-60 cells respectively, and luciferase assays were repeated four times.

MicroRNA hsa-miR-3148 and nontarget control (NC) mimics were synthesized by Thermo Fisher Scientific. To test the effect of miR-3148, HEK-293 cells plated in 96-well plates were transiently cotransfected with 100 ng of each reporter construct (psiCHECK-2 empty vector, rs3853839-G or -C allele constructs) and increasing concentrations (1, 6, 12 and 48 nM) of miR-3148 or nontarget control mimic using Lipofectamine 2000 reagent (Invitrogen), and luminescence was measured 24 hours after transfection. Each transfection was performed in quadruplicates and repeated three times. Luciferase activity of reporter vectors was compared using Student's *t* test.

### Assessment of allelic difference in RNA degradation rate

PBMCs isolated from EA healthy women with the GC genotype of rs3853839 (n = 7) were cultured in the absence or presence of 5 µg/mL ActD for 0, 2, 4, 6 and 24 hours. Using real-time PCR, we detected a decrease in total *TLR7* mRNA levels over time with ActD incubation, which confirmed the transcriptional inhibition by ActD and allowed for detection of allelic differences in mRNA degradation. The G/C allelic ratio in the cDNA and gDNA after treatment of PBMCs with or without ActD were determined by pyrosequencing and calculated using software PSQMA 2.1 (Biotage) as previously described [5]. The G/C allele ratio obtained in *TLR7* transcripts was normalized to that measured from gDNA of the same sample. A paired *t* test was used to compare the mean G/C allele ratio in *TLR7* transcripts in PBMCs treated with ActD or vehicle control at each time point.

### Statistical analysis

Associations of SNPs with SLE were assessed in each ancestral group under a logistic regression model adjusted for gender and the first three principal components estimated using AIMs. Conditional haplotype-based association tests were also performed by adjusting for gender and the first three principal components. The trans-ancestral meta-analysis was conducted on 40 genotyped and 14 imputed SNPs that were shared by the three ancestries with both a fixed and random-effects model. Homogeneity of odds ratios was evaluated using Cochrane's Q test. For each SNP, if the Cochran's Q test showed no evidence of genetic heterogeneity (*P*>0.05), a fixed-effects model was implemented; otherwise, a random-effects model was used. The Bonferroni corrected *P*-value threshold was adjusted to *P*<9.1×10^−4^ on the basis of the maximum number of tests across all populations (55 independent variants with r^2^<0.8). All analyses described above were performed using PLINK v1.07. Pairwised LD values shown in Figure 1 and Figure S1 were calculated using Haploview 4.2. Other data were analyzed using GraphPad Prism 4.0 software. A *P* value<0.05 was considered to be statistically significant.

## Supporting Information

Figure S1 Conditional haplotype-based association tests among seven SNPs within *TLR7-TLR8* region that show consistent association with SLE (*P*<0.05) in all three ancestral groups. (A) Trans-ancestry meta-analysis of 40 genotyped SNPs (circles) and 14 imputed SNPs (triangles) that are shared by the three ancestries using fixed and random model, respectively. The rectangle indicates the seven SNPs that show significant and consistent association with SLE in all three ancestral groups. Arrows identify the two strongest SNPs in the meta-analysis. The dashed line represents the significance level of 5×10^−8^. (B, C, D) Pairwised LD values (r^2^) of the seven SLE-associated SNPs, their allelic *P* value and *P* value after conditioning on the SNP shown as “–” are depicted in EA, AA and HS ancestry, respectively. ND represents that these two SNPs are non-distinguishable in the conditional test.
(TIF)

Figure S2 Representative dot plots and quantification of CD3^+^TLR7^+^ T cells, CD19^+^TLR7^+^ B cells and CD14^+^TLR7^+^ monocytes in PBMCs from healthy women (A, B) and men (C, D) carrying G or C allele of rs3853839 (n = 7 pairs GG or G vs. CC or C in each gender group). Numbers in upper quadrants indicate mean percentages of double positive cells in PBMCs.
(TIF)

Figure S3 Fluorescence-activated cell sorter (FACS) analysis of TLR8 staining. (A) FACS histograms show the log MFI values plotted against the cell counts for PBMCs in individuals carrying either G or C allele of rs3853839. Results are from 1 representative pair (GG or G vs. CC or C) of 7 in each gender group. (B) MFI of TLR8 expression in PBMCs is graphically depicted. Each symbol represents an individual and horizontal lines indicate mean ± SEM values. (C, D) Quantification of CD3^+^TLR8^+^ T cells, CD19^+^TLR8^+^ B cells and CD14^+^TLR8^+^ monocytes in PBMCs from healthy women and men carrying G or C allele of rs3853839, respectively.
(TIF)

Figure S4 Higher G/C allele ratio in cDNAs than in gDNAs from PBMCs of seven healthy EA women heterozygous for rs3853839.
(TIF)

Figure S5 The kinetics of *TLR7* mRNA levels in PBMCs after incubation with or without actinomycin D (ActD). PBMCs from heterozygous individuals (n = 7) were cultured in the absence or presence of 5 µg/mL actinomycin D for the indicated time, and then *TLR7* mRNA levels were measured by RT-PCR normalized to *RPLP0*. Data are presented as mean ± SEM at each time point and representative of two independent experiments with seven donors.
(TIF)

Table S1 Allelic associations of *TLR7/8* SNPs with SLE in European Americans (EA), African Americans (AA) and Amerindian/Hispanics (HS). Position of each SNP is based on GRch37/hg19. Seven SNPs that showed consistent association with SLE (*P*<0.05) in all 3 ancestral groups are highlighted in gray. The meta-analysis was performed with both fixed and random-effects model. If the Cochran's Q statistic showed no evidence of genetic heterogeneity (*P*>0.05), the *P* value and OR from a fixed effect model was applied. Otherwise, a random effect model was used. The finally applied meta *P* value and OR for each SNP are highlighted in bold. Abbreviation: G, genotype; I, imputed; OR, odds ratio; –, missing data.
(DOC)
