# Supplementary figures and images for: MicroRNA-3148 Modulates Allelic Expression of Toll-Like Receptor 7 Variant Associated with Systemic Lupus Erythematosus
Source: PLoS Genet. 2013 Feb 28;9(2):e1003336. doi: 10.1371/journal.pgen.1003336 (PMC3585142; doi:10.1371/journal.pgen.1003336)

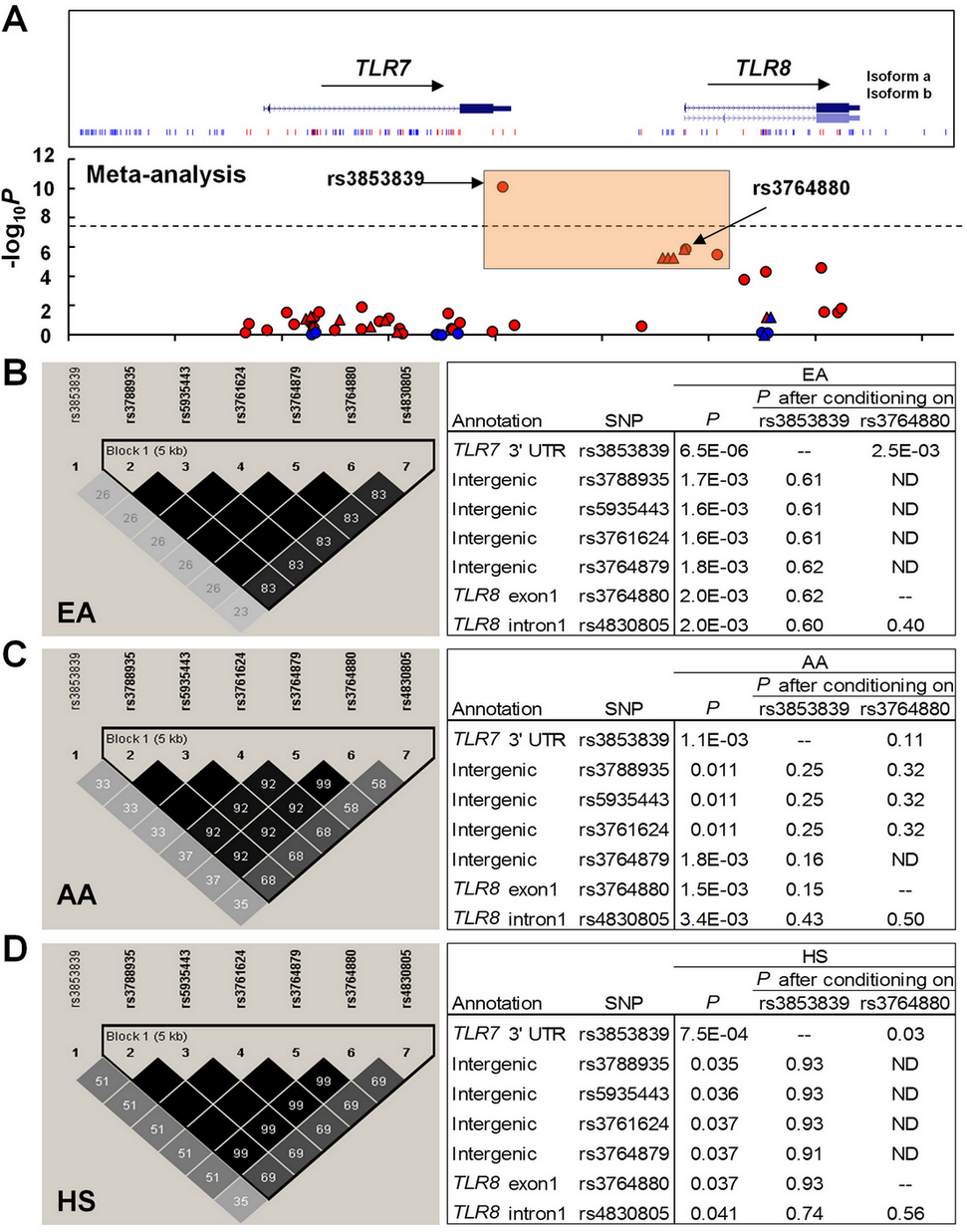

Supplement: Figure S1 — Conditional haplotype-based association tests among seven SNPs within TLR7-TLR8 region that show consistent association with SLE (P<0.05) in all three ancestral groups. (A) Trans-ancestry meta-analysis of 40 genotyped SNPs (circles) and 14 imputed SNPs (triangles) that are shared by the three ancestries using fixed and random model, respectively. The rectangle indicates the seven SNPs that show significant and consistent association with SLE in all three ancestral groups. Arrows identify the two strongest SNPs in the meta-analysis. The dashed line represents the significance level of 5×10^−8^. (B, C, D) Pairwised LD values (r2) of the seven SLE-associated SNPs, their allelic P value and P value after conditioning on the SNP shown as “–” are depicted in EA, AA and HS ancestry, respectively. ND represents that these two SNPs are non-distinguishable in the conditional test. (TIF) [file pgen.1003336.s001.tif]

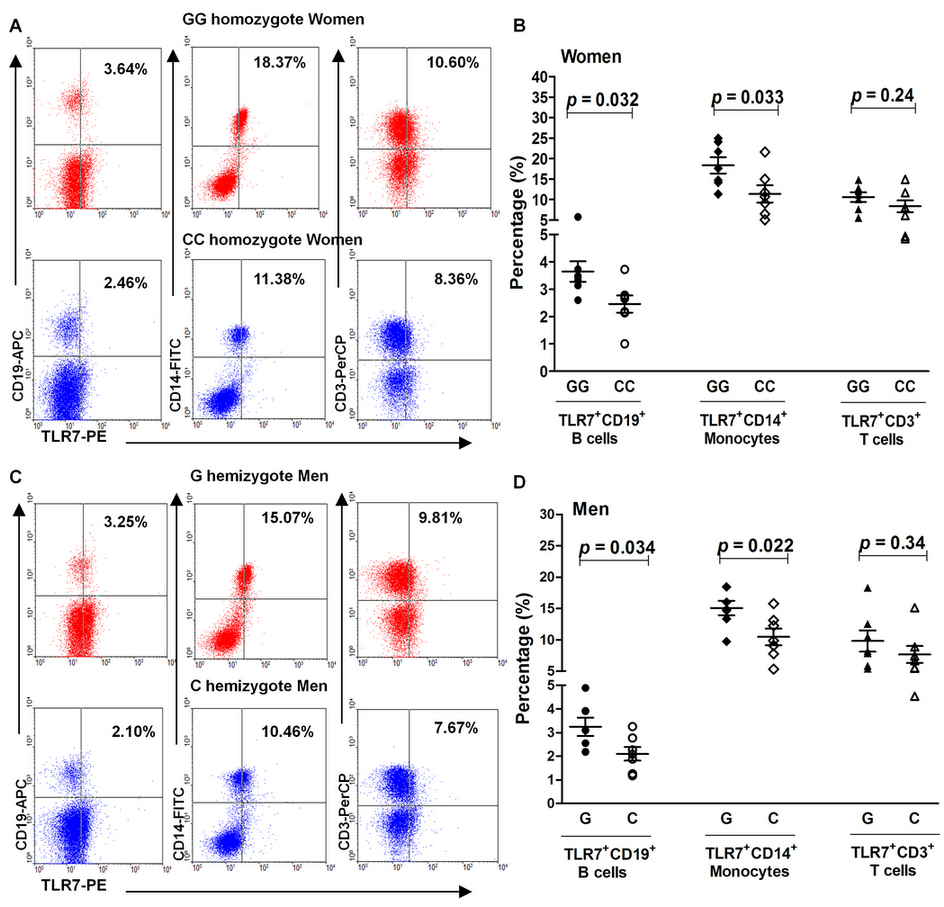

Supplement: Figure S2 — Representative dot plots and quantification of CD3+TLR7+ T cells, CD19+TLR7+ B cells and CD14+TLR7+ monocytes in PBMCs from healthy women (A, B) and men (C, D) carrying G or C allele of rs3853839 (n = 7 pairs GG or G vs. CC or C in each gender group). Numbers in upper quadrants indicate mean percentages of double positive cells in PBMCs. (TIF) [file pgen.1003336.s002.tif]

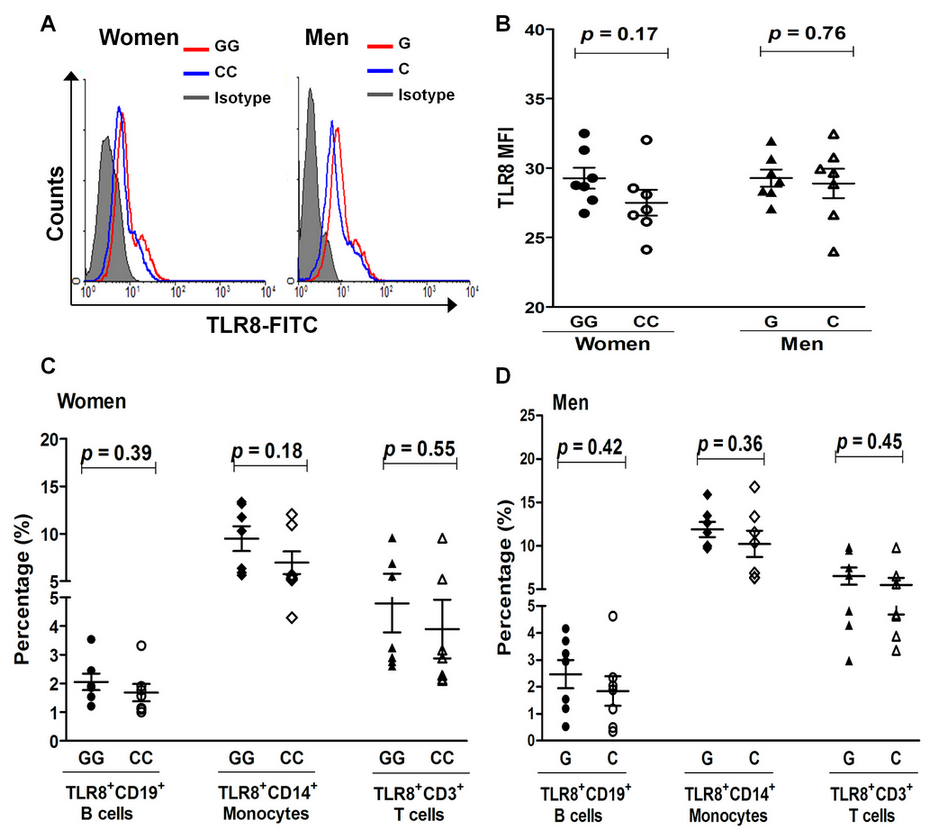

Supplement: Figure S3 — Fluorescence-activated cell sorter (FACS) analysis of TLR8 staining. (A) FACS histograms show the log MFI values plotted against the cell counts for PBMCs in individuals carrying either G or C allele of rs3853839. Results are from 1 representative pair (GG or G vs. CC or C) of 7 in each gender group. (B) MFI of TLR8 expression in PBMCs is graphically depicted. Each symbol represents an individual and horizontal lines indicate mean ± SEM values. (C, D) Quantification of CD3+TLR8+ T cells, CD19+TLR8+ B cells and CD14+TLR8+ monocytes in PBMCs from healthy women and men carrying G or C allele of rs3853839, respectively. (TIF) [file pgen.1003336.s003.tif]

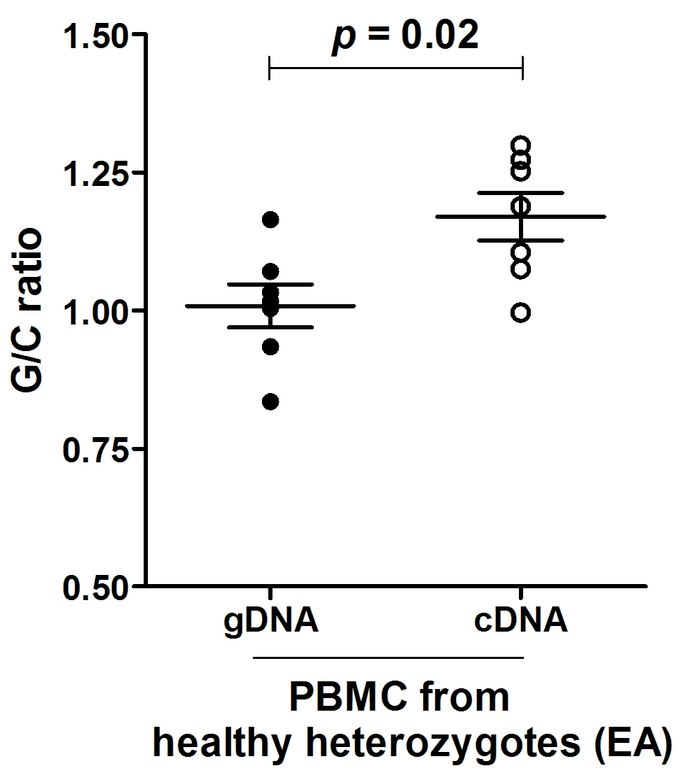

Supplement: Figure S4 — Higher G/C allele ratio in cDNAs than in gDNAs from PBMCs of seven healthy EA women heterozygous for rs3853839. (TIF) [file pgen.1003336.s004.tif]

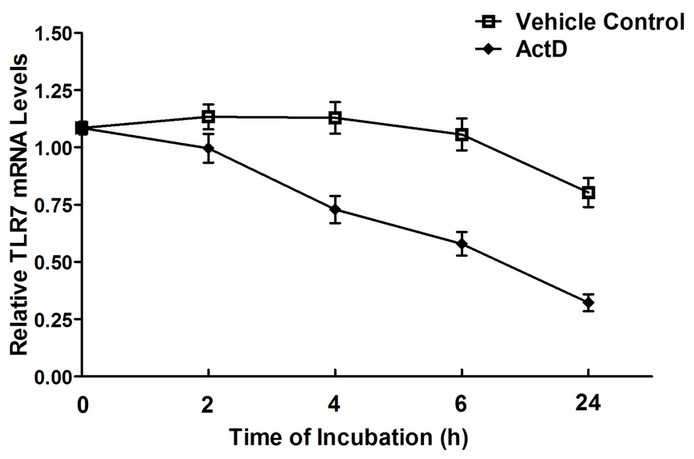

Supplement: Figure S5 — The kinetics of TLR7 mRNA levels in PBMCs after incubation with or without actinomycin D (ActD). PBMCs from heterozygous individuals (n = 7) were cultured in the absence or presence of 5 µg/mL actinomycin D for the indicated time, and then TLR7 mRNA levels were measured by RT-PCR normalized to RPLP0. Data are presented as mean ± SEM at each time point and representative of two independent experiments with seven donors. (TIF) [file pgen.1003336.s005.tif]
