# Supplementary material for: MicroRNA-3148 Modulates Allelic Expression of Toll-Like Receptor 7 Variant Associated with Systemic Lupus Erythematosus
Source: PLoS Genet. 2013 Feb 28;9(2):e1003336. doi: 10.1371/journal.pgen.1003336 (PMC3585142; doi:10.1371/journal.pgen.1003336)
Supplement: Table S1 — Allelic associations of TLR7/8 SNPs with SLE in European Americans (EA), African Americans (AA) and Amerindian/Hispanics (HS). Position of each SNP is based on GRch37/hg19. Seven SNPs that showed consistent association with SLE (P<0.05) in all 3 ancestral groups are highlighted in gray. The meta-analysis was performed with both fixed and random-effects model. If the Cochran's Q statistic showed no evidence of genetic heterogeneity (P>0.05), the P value and OR from a fixed effect model was applied. Otherwise, a random effect model was used. The finally applied meta P value and OR for each SNP are highlighted in bold. Abbreviation: G, genotype; I, imputed; OR, odds ratio; –, missing data. (DOC) [file pgen.1003336.s006.doc]

**Table S1**. Allelic associations of TLR7/8 SNPs with SLE in European Americans, African Americans and Amerindian/Hispanics

|  |  |  |  |  |  |  |  |  |  |  |  |  |  |  |  |  | Meta-analysis | | | |
| --- | --- | --- | --- | --- | --- | --- | --- | --- | --- | --- | --- | --- | --- | --- | --- | --- | --- | --- | --- | --- |
|  |  |  |  |  | EA | | | | AA | | | | HS | | | | Fixed Model | | Random Model | |
| Annotation | Type | SNP | Position | Allele | Case | Ctrl | P | OR | Case | Ctrl | P | OR | Case | Ctrl | P | OR | P | OR | P | OR |
| *TLR7* 5' upstream | I | rs6530571 | 12867907 | C | 18.3% | 16.8% | 0.014 | 1.12[1.02-1.24] | -- | -- | -- | -- | -- | -- | -- | -- | -- | -- | -- | -- |
| *TLR7* 5' upstream | I | rs7884128 | 12868006 | T | 18.3% | 16.8% | 0.014 | 1.12[1.02-1.24] | -- | -- | -- | -- | -- | -- | -- | -- | -- | -- | -- | -- |
| *TLR7* 5' upstream | I | rs5935421 | 12868147 | T | 18.3% | 16.8% | 0.014 | 1.12[1.02-1.24] | -- | -- | -- | -- | -- | -- | -- | -- | -- | -- | -- | -- |
| *TLR7* 5' upstream | I | rs5934044 | 12868434 | C | 18.3% | 16.8% | 0.014 | 1.12[1.02-1.24] | -- | -- | -- | -- | -- | -- | -- | -- | -- | -- | -- | -- |
| *TLR7* 5' upstream | I | rs5934045 | 12868521 | A | 17.1% | 15.7% | 0.014 | 1.13[1.03-1.25] | -- | -- | -- | -- | -- | -- | -- | -- | -- | -- | -- | -- |
| *TLR7* 5' upstream | I | rs140208335 | 12868703 | A | 5.3% | 5.2% | 0.671 | 1.04[0.88-1.22] | -- | -- | -- | -- | 7.9% | 7.7% | 0.843 | 1.02[0.81-1.29] | -- | -- | -- | -- |
| *TLR7* 5' upstream | I | rs151135117 | 12868914 | C | 18.3% | 16.8% | 0.014 | 1.12[1.02-1.24] | -- | -- | -- | -- | 18.0% | 17.7% | 0.494 | 1.06[0.90-1.25] | -- | -- | -- | -- |
| *TLR7* 5' upstream | I | rs141117346 | 12868992 | T | 18.3% | 16.8% | 0.014 | 1.12[1.02-1.24] | -- | -- | -- | -- | 18.0% | 17.7% | 0.484 | 1.06[0.90-1.25] | -- | -- | -- | -- |
| *TLR7* 5' upstream | I | rs146832267 | 12869156 | T | 5.3% | 5.2% | 0.671 | 1.04[0.88-1.22] | -- | -- | -- | -- | 7.7% | 7.3% | 0.769 | 1.04[0.82-1.32] | -- | -- | -- | -- |
| *TLR7* 5' upstream | I | rs146538469 | 12869348 | G | 18.3% | 16.8% | 0.014 | 1.13[1.02-1.24] | -- | -- | -- | -- | 18.0% | 17.7% | 0.491 | 1.06[0.90-1.25] | -- | -- | -- | -- |
| *TLR7* 5' upstream | I | rs140393376 | 12869742 | T | 12.2% | 10.9% | 0.011 | 1.16[1.04-1.29] | -- | -- | -- | -- | 7.2% | 8.4% | 0.609 | 0.94[0.75-1.19] | -- | -- | -- | -- |
| *TLR7* 5' upstream | I | rs6639231 | 12869984 | C | 18.3% | 16.8% | 0.014 | 1.13[1.02-1.24] | -- | -- | -- | -- | 18.0% | 17.7% | 0.490 | 1.06[0.90-1.25] | -- | -- | -- | -- |
| *TLR7* 5' upstream | I | rs6639232 | 12870213 | T | 17.2% | 15.7% | 9.5E-03 | 1.14[1.03-1.26] | -- | -- | -- | -- | 18.1% | 17.8% | 0.476 | 1.06[0.90-1.26] | -- | -- | -- | -- |
| *TLR7* 5' upstream | I | 12870732-D | 12870732 | C | 18.3% | 16.8% | 0.014 | 1.13[1.02-1.24] | -- | -- | -- | -- | 18.2% | 17.8% | 0.441 | 1.07[0.90-1.26] | -- | -- | -- | -- |
| *TLR7* 5' upstream | I | rs5935424 | 12870897 | C | 18.3% | 16.8% | 0.014 | 1.13[1.02-1.24] | -- | -- | -- | -- | 18.2% | 17.8% | 0.441 | 1.07[0.90-1.26] | -- | -- | -- | -- |
| *TLR7* 5' upstream | I | rs5935425 | 12870928 | T | 18.3% | 16.8% | 0.014 | 1.13[1.02-1.24] | -- | -- | -- | -- | 18.2% | 17.8% | 0.441 | 1.07[0.90-1.26] | -- | -- | -- | -- |
| *TLR7* 5' upstream | I | rs5935426 | 12871071 | A | 18.3% | 16.8% | 0.014 | 1.13[1.02-1.24] | -- | -- | -- | -- | 18.2% | 17.8% | 0.446 | 1.07[0.90-1.26] | -- | -- | -- | -- |
| *TLR7* 5' upstream | I | rs5935427 | 12871080 | T | 18.3% | 16.8% | 0.014 | 1.13[1.02-1.24] | -- | -- | -- | -- | 18.2% | 17.8% | 0.446 | 1.07[0.90-1.26] | -- | -- | -- | -- |
| *TLR7* 5' upstream | I | rs5935428 | 12871156 | G | 18.3% | 16.8% | 0.014 | 1.13[1.02-1.24] | -- | -- | -- | -- | 18.2% | 17.8% | 0.446 | 1.07[0.90-1.26] | -- | -- | -- | -- |
| *TLR7* 5' upstream | I | rs5935429 | 12871165 | A | 18.3% | 16.8% | 0.014 | 1.13[1.02-1.24] | -- | -- | -- | -- | 18.2% | 17.8% | 0.446 | 1.07[0.90-1.26] | -- | -- | -- | -- |
| *TLR7* 5' upstream | I | rs5935430 | 12871238 | C | 18.3% | 16.8% | 0.014 | 1.13[1.02-1.24] | -- | -- | -- | -- | 18.2% | 17.8% | 0.446 | 1.07[0.90-1.26] | -- | -- | -- | -- |
| *TLR7* 5' upstream | I | rs62589450 | 12871369 | T | 5.5% | 5.4% | 0.732 | 1.03[0.88-1.20] | -- | -- | -- | -- | 7.7% | 7.3% | 0.769 | 1.04[0.82-1.32] | -- | -- | -- | -- |
| *TLR7* 5' upstream | I | rs6530583 | 12871671 | A | 18.3% | 16.8% | 0.014 | 1.13[1.02-1.24] | -- | -- | -- | -- | 18.2% | 17.8% | 0.446 | 1.07[0.90-1.26] | -- | -- | -- | -- |
| *TLR7* 5' upstream | I | rs5935431 | 12872244 | G | 12.6% | 11.2% | 9.1E-03 | 1.16[1.04-1.29] | -- | -- | -- | -- | 9.8% | 10.0% | 0.643 | 1.05[0.85-1.30] | -- | -- | -- | -- |
| *TLR7* 5' upstream | I | rs62589455 | 12872556 | T | 5.5% | 5.4% | 0.732 | 1.03[0.88-1.20] | -- | -- | -- | -- | 8.0% | 7.7% | 0.762 | 1.04[0.82-1.31] | -- | -- | -- | -- |
| *TLR7* 5' upstream | I | rs6530584 | 12873044 | T | 12.6% | 11.2% | 0.011 | 1.16[1.03-1.29] | -- | -- | -- | -- | 9.8% | 10.0% | 0.613 | 1.06[0.85-1.31] | -- | -- | -- | -- |
| *TLR7* 5' upstream | I | rs6639233 | 12873161 | C | 18.4% | 16.8% | 0.014 | 1.13[1.03-1.24] | -- | -- | -- | -- | 18.2% | 17.9% | 0.494 | 1.06[0.90-1.25] | -- | -- | -- | -- |
| *TLR7* 5' upstream | I | rs2897826 | 12873411 | A | 5.5% | 5.4% | 0.732 | 1.03[0.88-1.20] | -- | -- | -- | -- | 8.0% | 7.7% | 0.762 | 1.04[0.82-1.31] | -- | -- | -- | -- |
| *TLR7* 5' upstream | I | rs2407990 | 12873545 | C | 18.4% | 16.8% | 0.014 | 1.13[1.02-1.24] | -- | -- | -- | -- | 18.2% | 17.9% | 0.494 | 1.06[0.90-1.25] | -- | -- | -- | -- |
| *TLR7* 5' upstream | I | 12874340-I | 12874340 | AACAG | 12.2% | 11.0% | 0.012 | 1.15[1.03-1.29] | -- | -- | -- | -- | 7.3% | 8.4% | 0.592 | 0.94[0.74-1.18] | -- | -- | -- | -- |
| *TLR7* 5' upstream | I | rs6641129 | 12876083 | T | 5.5% | 5.4% | 0.732 | 1.03[0.88-1.20] | -- | -- | -- | -- | 8.0% | 7.7% | 0.762 | 1.04[0.82-1.31] | -- | -- | -- | -- |
| *TLR7* 5' upstream | I | rs5935432 | 12876333 | A | 12.0% | 10.7% | 9.4E-03 | 1.16[1.04-1.30] | -- | -- | -- | -- | 7.7% | 8.8% | 0.668 | 0.95[0.76-1.20] | -- | -- | -- | -- |
| *TLR7* 5' upstream | I | rs12382242 | 12876880 | C | 5.5% | 5.4% | 0.732 | 1.03[0.88-1.20] | -- | -- | -- | -- | 8.0% | 7.7% | 0.762 | 1.04[0.82-1.31] | -- | -- | -- | -- |
| *TLR7* 5' upstream | I | rs138862332 | 12878028 | T | -- | -- | -- | -- | 26.7% | 27.3% | 0.696 | 0.98[0.87-1.10] | 9.8% | 9.9% | 0.552 | 1.07[0.86-1.32] | -- | -- | -- | -- |
| *TLR7* 5' upstream | I | rs144754651 | 12878442 | G | 12.6% | 11.2% | 9.1E-03 | 1.16[1.04-1.29] | -- | -- | -- | -- | 10.0% | 10.1% | 0.562 | 1.07[0.86-1.32] | -- | -- | -- | -- |
| *TLR7* 5' upstream | I | rs188109872 | 12878452 | G | 12.2% | 11.0% | 0.012 | 1.15[1.03-1.29] | -- | -- | -- | -- | 7.3% | 8.4% | 0.621 | 0.94[0.75-1.19] | -- | -- | -- | -- |
| *TLR7* 5' upstream | I | rs146702667 | 12880060 | C | 6.3% | 6.0% | 0.428 | 1.06[0.92-1.23] | -- | -- | -- | -- | 8.0% | 7.7% | 0.762 | 1.04[0.82-1.31] | -- | -- | -- | -- |
| *TLR7* 5' upstream | I | rs182780259 | 12880553 | T | 6.3% | 6.0% | 0.428 | 1.06[0.92-1.23] | -- | -- | -- | -- | 8.0% | 7.7% | 0.762 | 1.04[0.82-1.31] | -- | -- | -- | -- |
| *TLR7* 5' upstream | I | rs143996738 | 12880774 | T | 12.2% | 10.9% | 0.010 | 1.16[1.04-1.29] | -- | -- | -- | -- | 7.6% | 8.6% | 0.748 | 0.96[0.77-1.21] | -- | -- | -- | -- |
| *TLR7* 5' upstream | I | rs146414410 | 12881202 | A | 12.2% | 10.9% | 0.010 | 1.16[1.04-1.29] | -- | -- | -- | -- | 7.3% | 8.4% | 0.621 | 0.94[0.75-1.19] | -- | -- | -- | -- |
| *TLR7* 5' upstream | I | rs151323749 | 12881361 | C | 6.3% | 6.0% | 0.428 | 1.06[0.92-1.23] | -- | -- | -- | -- | 8.0% | 7.7% | 0.762 | 1.04[0.82-1.31] | -- | -- | -- | -- |
| *TLR7* 5' upstream | G | rs2897827 | 12883546 | T | 6.4% | 6.1% | 0.394 | 1.07[0.92-1.23] | 9.3% | 9.6% | 0.705 | 0.97[0.82-1.15] | 8.1% | 7.8% | 0.854 | 1.02[0.81-1.29] | **0.661** | **1.02** | 0.661 | 1.02 |
| *TLR7* 5' upstream | G | rs5935436 | 12883891 | T | 13.5% | 12.4% | 0.039 | 1.12[1.01-1.24] | 27.9% | 28.3% | 0.766 | 0.98[0.88-1.10] | 10.9% | 11.1% | 0.621 | 1.05[0.86-1.29] | **0.168** | **1.05** | 0.262 | 1.05 |
| *TLR7* intron1 | G | rs2302267 | 12885578 | G | 6.4% | 6.3% | 0.573 | 1.04[0.90-1.20] | 2.6% | 2.4% | 0.947 | 1.01[0.74-1.38] | 8.3% | 7.7% | 0.594 | 1.07[0.84-1.35] | **0.463** | **1.04** | 0.463 | 1.04 |
| *TLR7* intron2 | G | rs5741880 | 12887416 | T | 13.5% | 12.2% | 0.016 | 1.14[1.02-1.27] | 41.8% | 41.3% | 0.438 | 1.04[0.94-1.15] | 11.7% | 11.9% | 0.741 | 1.03[0.85-1.26] | **0.029** | **1.08** | 0.029 | 1.08 |
| *TLR7* intron2 | G | rs1634323 | 12888127 | G | 11.2% | 10.3% | 0.049 | 1.12[1.00-1.26] | 13.9% | 14.3% | 0.949 | 1.00[0.86-1.15] | 6.3% | 7.0% | 0.882 | 0.98[0.76-1.26] | **0.173** | **1.06** | 0.183 | 1.06 |
| *TLR7* intron2 | I | rs1731477 | 12889246 | T | 10.2% | 9.2% | 0.039 | 1.14[1.01-1.28] | 4.0% | 3.8% | 0.643 | 1.06[0.82-1.39] | 4.8% | 5.7% | 0.655 | 0.94[0.71-1.24] | **0.077** | **1.10** | 0.077 | 1.10 |
| *TLR7* intron2 | I | rs1731478 | 12889666 | T | 10.2% | 9.2% | 0.041 | 1.13[1.01-1.28] | 3.2% | 2.8% | 0.339 | 1.16[0.86-1.56] | 4.8% | 5.6% | 0.710 | 0.95[0.72-1.26] | **0.051** | **1.11** | 0.051 | 1.11 |
| *TLR7* intron2 | G | rs5743733 | 12889708 | G | 11.1% | 10.2% | 0.053 | 1.12[1.00-1.25] | 12.7% | 12.2% | 0.560 | 1.05[0.90-1.22] | 7.9% | 9.1% | 0.467 | 0.92[0.74-1.15] | **0.138** | **1.07** | 0.235 | 1.06 |
| *TLR7* intron2 | G | rs179021 | 12889763 | G | 19.5% | 20.2% | 0.143 | 0.94[0.86-1.02] | 6.1% | 7.1% | 0.164 | 0.87[0.71-1.06] | 19.3% | 16.8% | 0.037 | 1.19[1.01-1.40] | 0.437 | 0.97 | **0.903** | **0.99** |
| *TLR7* intron2 | I | rs5743734 | 12889809 | T | -- | -- | -- | -- | 7.2% | 6.7% | 0.390 | 1.09[0.89-1.34] | -- | -- | -- | -- | -- | -- | -- | -- |
| *TLR7* intron2 | I | rs179020 | 12889857 | A | 22.7% | 22.2% | 0.387 | 1.04[0.95-1.13] | 12.4% | 12.4% | 0.998 | 1.00[0.86-1.17] | 15.6% | 14.9% | 0.241 | 1.11[0.93-1.32] | **0.246** | **1.04** | 0.246 | 1.04 |
| *TLR7* intron2 | G | rs179019 | 12889969 | A | 22.8% | 22.4% | 0.446 | 1.03[0.95-1.12] | 12.4% | 12.5% | 0.951 | 1.00[0.85-1.16] | 15.6% | 15.0% | 0.241 | 1.11[0.93-1.32] | **0.296** | **1.04** | 0.296 | 1.04 |
| *TLR7* intron2 | G | rs1634322 | 12890027 | A | 21.4% | 19.5% | 3.8E-03 | 1.14[1.04-1.24] | 30.6% | 30.7% | 0.947 | 1.00[0.90-1.12] | 14.0% | 16.0% | 0.460 | 0.94[0.79-1.12] | **0.061** | **1.06** | 0.502 | 1.04 |
| *TLR7* intron2 | G | rs179018 | 12890150 | C | 20.4% | 21.1% | 0.137 | 0.94[0.86-1.02] | 11.9% | 12.1% | 0.907 | 0.99[0.85-1.16] | 20.6% | 17.4% | 0.010 | 1.24[1.05-1.45] | 0.888 | 1.00 | **0.662** | **1.04** |
| *TLR7* intron2 | I | rs5743735 | 12890244 | C | -- | -- | -- | -- | 3.6% | 3.8% | 0.506 | 0.92[0.70-1.19] | -- | -- | -- | -- | -- | -- | -- | -- |
| *TLR7* intron2 | G | rs1731479 | 12890453 | T | 21.3% | 19.5% | 3.8E-03 | 1.14[1.04-1.24] | 26.0% | 25.9% | 0.669 | 1.03[0.91-1.15] | 13.6% | 15.3% | 0.674 | 0.96[0.81-1.15] | **0.027** | **1.08** | 0.233 | 1.06 |
| *TLR7* intron2 | I | rs5743737 | 12891021 | G | -- | -- | -- | -- | 7.9% | 8.3% | 0.836 | 0.98[0.81-1.18] | -- | -- | -- | -- | -- | -- | -- | -- |
| *TLR7* intron2 | I | rs5743738 | 12891708 | C | -- | -- | -- | -- | 3.6% | 3.7% | 0.538 | 0.92[0.71-1.20] | -- | -- | -- | -- | -- | -- | -- | -- |
| *TLR7* intron2 | I | rs5743739 | 12891889 | G | -- | -- | -- | -- | 7.7% | 7.1% | 0.296 | 1.11[0.91-1.35] | -- | -- | -- | -- | -- | -- | -- | -- |
| *TLR7* intron2 | G | rs5743740 | 12891960 | G | 6.3% | 6.4% | 0.922 | 0.99[0.86-1.15] | 28.9% | 30.0% | 0.252 | 0.94[0.84-1.05] | 10.3% | 9.6% | 0.712 | 1.04[0.84-1.29] | **0.451** | **0.97** | 0.451 | 0.97 |
| *TLR7* intron2 | I | rs192843990 | 12892305 | A | 6.0% | 5.2% | 0.020 | 1.21[1.03-1.41] | -- | -- | -- | -- | 3.2% | 3.2% | 0.516 | 1.13[0.79-1.61] | -- | -- | -- | -- |
| *TLR7* intron2 | I | rs1638594 | 12892394 | A | 10.1% | 9.2% | 0.068 | 1.12[0.99-1.26] | 3.8% | 3.5% | 0.365 | 1.13[0.86-1.49] | 4.7% | 5.6% | 0.609 | 0.93[0.70-1.23] | **0.089** | **1.09** | 0.089 | 1.09 |
| *TLR7* intron2 | I | rs5743742 | 12892648 | A | -- | -- | -- | -- | 6.1% | 5.6% | 0.429 | 1.09[0.88-1.36] | -- | -- | -- | -- | -- | -- | -- | -- |
| *TLR7* intron2 | I | rs5743744 | 12893700 | C | -- | -- | -- | -- | 11.3% | 11.3% | 0.902 | 1.01[0.86-1.18] | -- | -- | -- | -- | -- | -- | -- | -- |
| *TLR7* intron2 | I | rs148570346 | 12893724 | T | 4.9% | 4.4% | 0.096 | 1.15[0.97-1.36] | -- | -- | -- | -- | -- | -- | -- | -- | -- | -- | -- | -- |
| *TLR7* intron2 | I | rs5743747 | 12893835 | A | -- | -- | -- | -- | 13.7% | 13.5% | 0.750 | 1.02[0.88-1.19] | -- | -- | -- | -- | -- | -- | -- | -- |
| *TLR7* intron2 | I | rs111487020 | 12894017 | A | -- | -- | -- | -- | 7.9% | 7.2% | 0.259 | 1.12[0.92-1.36] | -- | -- | -- | -- | -- | -- | -- | -- |
| *TLR7* intron2 | I | rs5743748 | 12894303 | T | -- | -- | -- | -- | 13.7% | 13.4% | 0.675 | 1.03[0.89-1.20] | -- | -- | -- | -- | -- | -- | -- | -- |
| *TLR7* intron2 | G | rs179016 | 12894442 | C | 37.5% | 37.1% | 0.893 | 1.01[0.93-1.08] | 80.0% | 80.6% | 0.588 | 0.97[0.85-1.10] | 38.8% | 35.6% | 0.022 | 1.16[1.02-1.33] | **0.389** | **1.03** | 0.482 | 1.04 |
| *TLR7* intron2 | G | rs5743749 | 12894491 | A | 6.2% | 7.6% | 5.1E-03 | 0.82[0.71-0.94] | 1.0% | 0.9% | 0.505 | 1.20[0.70-2.05] | 3.4% | 4.1% | 0.660 | 0.93[0.66-1.30] | **0.012** | **0.85** | 0.032 | 0.86 |
| *TLR7* intron2 | I | rs5743750 | 12894827 | T | -- | -- | -- | -- | 13.7% | 13.4% | 0.697 | 1.03[0.89-1.19] | -- | -- | -- | -- | -- | -- | -- | -- |
| *TLR7* intron2 | I | rs5743751 | 12894860 | G | -- | -- | -- | -- | 3.5% | 3.7% | 0.531 | 0.92[0.71-1.20] | -- | -- | -- | -- | -- | -- | -- | -- |
| *TLR7* intron2 | I | rs7886759 | 12895122 | A | -- | -- | -- | -- | 7.2% | 6.8% | 0.436 | 1.08[0.89-1.32] | -- | -- | -- | -- | -- | -- | -- | -- |
| *TLR7* intron2 | I | rs1638595 | 12895292 | A | 9.7% | 9.0% | 0.121 | 1.10[0.97-1.25] | 23.7% | 24.0% | 0.855 | 1.01[0.90-1.14] | 6.2% | 6.8% | 0.936 | 0.99[0.77-1.28] | **0.263** | **1.05** | 0.263 | 1.05 |
| *TLR7* intron2 | G | rs1634321 | 12895325 | T | -- | -- | -- | -- | 26.6% | 27.1% | 0.937 | 1.00[0.89-1.11] | 3.2% | 3.2% | 0.709 | 1.07[0.75-1.52] | -- | -- | -- | -- |
| *TLR7* intron2 | G | rs1638596 | 12896110 | C | 10.3% | 9.4% | 0.064 | 1.12[0.99-1.26] | 5.1% | 4.7% | 0.425 | 1.10[0.87-1.39] | 4.9% | 6.0% | 0.450 | 0.90[0.69-1.18] | **0.109** | **1.08** | 0.136 | 1.08 |
| *TLR7* intron2 | I | rs5743753 | 12896605 | A | -- | -- | -- | -- | 4.0% | 4.3% | 0.948 | 0.99[0.77-1.28] | -- | -- | -- | -- | -- | -- |  |  |
| *TLR7* intron2 | I | rs1638597 | 12896661 | T | 10.2% | 9.4% | 0.076 | 1.12[0.99-1.26] | 34.6% | 34.0% | 0.391 | 1.05[0.94-1.17] | 6.5% | 7.2% | 0.839 | 0.97[0.76-1.25] | **0.095** | **1.07** | 0.095 | 1.07 |
| *TLR7* intron2 | I | 12896868-D | 12896868 | A | -- | -- | -- | -- | 13.4% | 12.9% | 0.567 | 1.04[0.90-1.21] | -- | -- | -- | -- | -- | -- | -- | -- |
| *TLR7* intron2 | I | 12896871-I | 12896871 | TG | -- | -- | -- | -- | 13.5% | 13.1% | 0.622 | 1.04[0.89-1.20] | -- | -- | -- | -- | -- | -- | -- | -- |
| *TLR7* intron2 | I | 12896872-D | 12896872 | A | -- | -- | -- | -- | 13.5% | 13.1% | 0.622 | 1.04[0.89-1.20] | -- | -- | -- | -- | -- | -- | -- | -- |
| *TLR7* intron2 | I | 12896876-I | 12896876 | TGA | -- | -- | -- | -- | 13.5% | 13.1% | 0.604 | 1.04[0.90-1.21] | -- | -- | -- | -- | -- | -- | -- | -- |
| *TLR7* intron2 | G | rs1634320 | 12897021 | T | 10.2% | 9.3% | 0.060 | 1.12[1.00-1.27] | 4.1% | 3.7% | 0.313 | 1.14[0.88-1.49] | 4.8% | 5.7% | 0.639 | 0.94[0.71-1.24] | **0.070** | **1.10** | 0.070 | 1.10 |
| *TLR7* intron2 | I | 12897766-D | 12897766 | C | 10.3% | 9.4% | 0.071 | 1.12[0.99-1.26] | 51.2% | 52.6% | 0.409 | 0.96[0.87-1.06] | 8.8% | 9.3% | 0.971 | 1.00[0.80-1.24] | **0.621** | **1.02** | 0.680 | 1.02 |
| *TLR7* intron2 | G | rs1620233 | 12898016 | T | 10.9% | 9.9% | 0.033 | 1.14[1.01-1.28] | 48.9% | 49.9% | 0.592 | 0.97[0.88-1.08] | 9.0% | 9.3% | 0.919 | 1.01[0.81-1.26] | **0.344** | **1.04** | 0.485 | 1.04 |
| *TLR7* intron2 | I | rs5743761 | 12898072 | G | -- | -- | -- | -- | 13.3% | 13.2% | 0.834 | 1.02[0.88-1.18] | -- | -- | -- | -- | -- | -- | -- | -- |
| *TLR7* intron2 | G | rs5743763 | 12898293 | A | 0.2% | 0.2% | 0.872 | 1.07[0.49-2.33] | 21.7% | 22.0% | 0.778 | 0.98[0.87-1.11] | 1.8% | 1.9% | 0.862 | 0.96[0.60-1.54] | **0.773** | **0.98** | 0.773 | 0.98 |
| *TLR7* intron2 | I | rs148171431 | 12898902 | A | -- | -- | -- | -- | 1.5% | 1.7% | 0.464 | 0.86[0.58-1.29] | -- | -- | -- | -- | -- | -- | -- | -- |
| *TLR7* intron2 | I | rs141988820 | 12898916 | T | 4.9% | 4.5% | 0.113 | 1.15[0.97-1.36] | -- | -- | -- | -- | 3.2% | 3.2% | 0.499 | 1.13[0.79-1.62] | -- | -- | -- | -- |
| *TLR7* intron2 | I | rs138928238 | 12899010 | T | -- | -- | -- | -- | 3.5% | 3.6% | 0.589 | 0.93[0.71-1.21] | -- | -- | -- | -- | -- | -- | -- | -- |
| *TLR7* intron2 | I | rs142136447 | 12899061 | A | -- | -- | -- | -- | 3.5% | 3.6% | 0.589 | 0.93[0.71-1.21] | -- | -- | -- | -- | -- | -- | -- | -- |
| *TLR7* intron2 | I | rs5743765 | 12899271 | C | 5.5% | 5.6% | 0.964 | 1.00[0.86-1.17] | -- | -- | -- | -- | 7.8% | 7.1% | 0.588 | 1.07[0.84-1.37] | -- | -- | -- | -- |
| *TLR7* intron2 | I | rs5743766 | 12899307 | C | -- | -- | -- | -- | 4.0% | 3.8% | 0.962 | 0.99[0.77-1.28] | -- | -- | -- | -- | -- | -- | -- | -- |
| *TLR7* intron2 | I | 12899485-D | 12899485 | G | -- | -- | -- | -- | 3.5% | 3.5% | 0.794 | 0.96[0.74-1.27] | -- | -- | -- | -- | -- | -- | -- | -- |
| *TLR7* intron2 | I | 12899561-D | 12899561 | T | -- | -- | -- | -- | 3.4% | 3.4% | 0.964 | 1.01[0.76-1.33] | -- | -- | -- | -- | -- | -- | -- | -- |
| *TLR7* intron2 | I | 12899626-D | 12899626 | A | 9.9% | 9.4% | 0.211 | 1.08[0.96-1.22] | -- | -- | -- | -- | 4.9% | 5.9% | 0.600 | 0.93[0.70-1.23] | -- | -- | -- | -- |
| *TLR7* intron2 | I | 12899696-D | 12899696 | A | -- | -- | -- | -- | -- | -- | -- | -- | 4.8% | 5.7% | 0.655 | 0.94[0.71-1.24] | -- | -- | -- | -- |
| *TLR7* intron2 | I | rs5743770 | 12900574 | A | -- | -- | -- | -- | 7.2% | 7.0% | 0.688 | 1.04[0.85-1.28] | -- | -- | -- | -- | -- | -- | -- | -- |
| *TLR7* intron2 | I | rs5743771 | 12900915 | A | -- | -- | -- | -- | 7.0% | 6.8% | 0.718 | 1.04[0.85-1.27] | -- | -- | -- | -- | -- | -- | -- | -- |
| *TLR7* intron2 | G | rs179013 | 12901471 | A | 20.0% | 20.7% | 0.136 | 0.94[0.86-1.02] | 10.3% | 11.5% | 0.238 | 0.91[0.77-1.07] | 19.6% | 17.2% | 0.047 | 1.18[1.00-1.39] | 0.398 | 0.97 | **0.927** | **0.99** |
| *TLR7* intron2 | G | rs179012 | 12901562 | A | 29.0% | 29.7% | 0.244 | 0.95[0.88-1.03] | 71.3% | 73.4% | 0.120 | 0.91[0.82-1.02] | 33.8% | 29.7% | 9.0E-03 | 1.20[1.05-1.37] | 0.578 | 0.98 | **0.912** | **1.01** |
| *TLR7* intron2 | G | rs179011 | 12901960 | T | 20.6% | 21.3% | 0.178 | 0.94[0.86-1.03] | 39.5% | 41.7% | 0.127 | 0.92[0.84-1.02] | 22.2% | 19.4% | 0.031 | 1.19[1.02-1.39] | 0.305 | 0.97 | **0.946** | **1.00** |
| *TLR7* intron2 | I | rs5743775 | 12902336 | T | -- | -- | -- | -- | 7.1% | 6.1% | 0.070 | 1.21[0.99-1.48] | -- | -- | -- | -- | -- | -- | -- | -- |
| *TLR7* intron2 | G | rs5743776 | 12902545 | T | 0.1% | 0.1% | 0.814 | 0.86[0.24-3.06] | 7.2% | 6.1% | 0.073 | 1.20[0.98-1.47] | 1.1% | 0.5% | 0.063 | 2.19[0.96-5.01] | **0.034** | **1.23** | 0.110 | 1.27 |
| *TLR7* intron2 | I | rs148118720 | 12902773 | A | -- | -- | -- | -- | 1.7% | 1.3% | 0.125 | 1.39[0.91-2.10] | -- | -- | -- | -- | -- | -- | -- | -- |
| *TLR7* intron2 | G | rs179010 | 12902885 | T | 30.5% | 29.9% | 0.207 | 1.05[0.97-1.13] | 15.3% | 14.9% | 0.766 | 1.02[0.89-1.18] | 14.6% | 16.9% | 0.513 | 0.94[0.79-1.12] | **0.348** | **1.03** | 0.348 | 1.03 |
| *TLR7* intron2 | G | rs1634319 | 12903035 | C | 9.5% | 8.6% | 0.077 | 1.12[0.99-1.26] | 28.7% | 29.2% | 0.539 | 0.97[0.87-1.08] | 8.6% | 8.9% | 0.661 | 1.05[0.84-1.31] | **0.409** | **1.03** | 0.474 | 1.04 |
| *TLR7* intron2 | G | rs179009 | 12903480 | G | 20.6% | 21.4% | 0.109 | 0.93[0.85-1.02] | 15.8% | 16.7% | 0.335 | 0.94[0.82-1.07] | 30.8% | 25.7% | 2.0E-03 | 1.25[1.09-1.45] | 0.797 | 0.99 | **0.791** | **1.02** |
| *TLR7* exon3 | G | rs179008 | 12903659 | T | 20.4% | 21.2% | 0.084 | 0.93[0.85-1.01] | 11.7% | 13.2% | 0.116 | 0.88[0.76-1.03] | 19.4% | 17.8% | 0.150 | 1.13[0.96-1.33] | **0.143** | **0.95** | 0.581 | 0.97 |
| *TLR7* exon3 | G | rs5741881 | 12906714 | G | 0.2% | 0.0% | 0.051 | 4.56[0.99-21.01] | 20.6% | 20.8% | 0.401 | 0.95[0.84-1.07] | 1.8% | 1.6% | 0.758 | 1.08[0.66-1.75] | **0.559** | **0.97** | 0.664 | 1.09 |
| *TLR7* 3'UTR | G | rs3853839 | 12907658 | G | 20.3% | 17.2% | 6.5E-06 | 1.23[1.13-1.35] | 19.8% | 16.7% | 1.1E-03 | 1.24[1.09-1.41] | 44.8% | 37.3% | 7.5E-04 | 1.26[1.10-1.43] | **7.5E-11** | **1.24** | 7.5E-11 | 1.24 |
| Intergenic | G | rs850633 | 12908782 | A | 10.9% | 10.5% | 0.553 | 1.04[0.92-1.16] | 22.9% | 22.1% | 0.311 | 1.06[0.94-1.20] | 8.2% | 8.6% | 0.536 | 1.07[0.86-1.35] | **0.202** | **1.05** | 0.202 | 1.05 |
| Intergenic | I | rs6641138 | 12920489 | T | 29.3% | 27.1% | 1.5E-03 | 1.14[1.05-1.23] | -- | -- | -- | -- | -- | -- | -- | -- | -- | -- | -- | -- |
| Intergenic | G | rs178994 | 12920700 | G | 11.3% | 11.8% | 0.307 | 0.94[0.85-1.05] | 1.6% | 1.7% | 0.934 | 0.98[0.66-1.46] | 5.0% | 6.3% | 0.495 | 0.91[0.70-1.19] | **0.236** | **0.94** | 0.236 | 0.94 |
| Intergenic | I | rs3788935 | 12922659 | G | 28.8% | 26.7% | 1.7E-03 | 1.14[1.05-1.23] | 25.9% | 23.5% | 0.011 | 1.17[1.04-1.31] | 48.6% | 43.7% | 0.035 | 1.15[1.01-1.30] | **5.4E-06** | **1.15** | 5.4E-06 | 1.15 |
| Intergenic | I | rs5935442 | 12923109 | T | 29.4% | 27.3% | 2.5E-03 | 1.13[1.04-1.23] | -- | -- | -- | -- | 48.6% | 43.7% | 0.036 | 1.15[1.01-1.30] | -- | -- | -- | -- |
| Intergenic | I | rs5935443 | 12923197 | T | 28.9% | 26.7% | 1.6E-03 | 1.14[1.05-1.23] | 25.9% | 23.5% | 0.011 | 1.17[1.04-1.31] | 48.6% | 43.7% | 0.036 | 1.15[1.01-1.30] | **5.5E-06** | **1.15** | 5.5E-06 | 1.15 |
| Intergenic | I | rs5744041 | 12923555 | T | -- | -- | -- | -- | 2.0% | 2.1% | 0.757 | 0.95[0.66-1.35] | -- | -- | -- | -- | -- | -- | -- | -- |
| Intergenic | I | rs3761624 | 12923681 | G | 28.9% | 26.7% | 1.6E-03 | 1.14[1.05-1.23] | 25.9% | 23.5% | 0.011 | 1.17[1.04-1.31] | 48.6% | 43.7% | 0.037 | 1.15[1.01-1.30] | **5.5E-06** | **1.15** | 5.5E-06 | 1.15 |
| Intergenic | I | rs3764879 | 12924697 | G | 28.9% | 26.7% | 1.8E-03 | 1.14[1.05-1.23] | 29.1% | 26.0% | 1.8E-03 | 1.20[1.07-1.34] | 48.6% | 43.7% | 0.037 | 1.15[1.01-1.30] | **1.4E-06** | **1.15** | 1.4E-06 | 1.15 |
| *TLR8* exon1 | G | rs3764880 | 12924826 | G | 28.9% | 26.8% | 2.0E-03 | 1.13[1.05-1.23] | 29.2% | 26.0% | 1.5E-03 | 1.20[1.07-1.34] | 48.7% | 43.8% | 0.037 | 1.15[1.01-1.30] | **1.3E-06** | **1.15** | 1.3E-06 | 1.15 |
| *TLR8* intron1 | I | rs142934414 | 12926375 | A | -- | -- | -- | -- | 2.0% | 2.0% | 0.890 | 0.98[0.68-1.39] | -- | -- | -- | -- | -- | -- | -- | -- |
| *TLR8* intron1 | I | rs146105516 | 12926824 | A | -- | -- | -- | -- | 2.1% | 1.4% | 0.059 | 1.46[0.99-2.16] | -- | -- | -- | -- | -- | -- | -- | -- |
| *TLR8* intron1 | G | rs4830805 | 12927759 | A | 26.4% | 24.2% | 2.0E-03 | 1.14[1.05-1.23] | 24.5% | 21.5% | 3.4E-03 | 1.19[1.06-1.35] | 40.7% | 36.5% | 0.041 | 1.15[1.01-1.30] | **3.1E-06** | **1.15** | 3.1E-06 | 1.15 |
| *TLR8* intron1 | G | rs3827469 | 12930303 | G | 19.8% | 17.7% | 1.6E-03 | 1.16[1.06-1.27] | 13.4% | 12.9% | 0.626 | 1.04[0.89-1.20] | 33.2% | 28.6% | 0.014 | 1.19[1.04-1.36] | **1.6E-04** | **1.14** | 1.6E-04 | 1.14 |
| *TLR8* intron1 | G | rs4830806 | 12931972 | T | 41.6% | 39.2% | 5.5E-03 | 1.11[1.03-1.19] | 67.0% | 68.0% | 0.209 | 0.93[0.84-1.04] | 64.9% | 61.7% | 0.686 | 1.03[0.90-1.17] | 0.104 | 1.05 | **0.647** | **1.03** |
| *TLR8* intron1 | I | rs6639237 | 12932074 | C | -- | -- | -- | -- | 2.6% | 2.4% | 0.437 | 1.14[0.82-1.56] | -- | -- | -- | -- | -- | -- | -- | -- |
| *TLR8* intron1 | I | rs6639238 | 12932142 | T | -- | -- | -- | -- | 2.6% | 2.4% | 0.437 | 1.14[0.82-1.56] | -- | -- | -- | -- | -- | -- | -- | -- |
| *TLR8* intron1 | I | rs4830807 | 12932197 | C | 41.6% | 39.2% | 5.3E-03 | 1.11[1.03-1.19] | 60.5% | 61.3% | 0.256 | 0.94[0.85-1.05] | 64.2% | 61.2% | 0.806 | 1.02[0.89-1.16] | 0.109 | 1.05 | **0.635** | **1.03** |
| *TLR8* intron1 | G | rs4830808 | 12932334 | T | 20.6% | 18.3% | 1.0E-03 | 1.16[1.06-1.28] | 8.4% | 7.8% | 0.296 | 1.10[0.92-1.33] | 32.7% | 28.6% | 0.026 | 1.17[1.02-1.34] | **4.8E-05** | **1.16** | 4.8E-05 | 1.16 |
| *TLR8* intron1 | I | rs1013150 | 12932441 | A | 21.9% | 21.7% | 0.863 | 1.01[0.92-1.10] | 50.2% | 51.8% | 0.081 | 0.91[0.83-1.01] | 31.7% | 33.4% | 0.024 | 0.85[0.75-0.98] | **0.059** | **0.94** | 0.145 | 0.93 |
| *TLR8* intron1 | G | rs1013151 | 12932531 | T | 41.5% | 39.2% | 5.7E-03 | 1.11[1.03-1.19] | 64.4% | 65.5% | 0.133 | 0.92[0.83-1.03] | 64.6% | 61.2% | 0.567 | 1.04[0.91-1.18] | 0.125 | 1.04 | **0.692** | **1.02** |
| *TLR8* intron1 | I | rs5744054 | 12932721 | T | 5.5% | 5.4% | 0.767 | 1.02[0.87-1.20] | -- | -- | -- | -- | -- | -- | -- | -- | -- | -- | -- | -- |
| *TLR8* intron1 | I | rs5741884 | 12932811 | A | 41.5% | 39.2% | 5.5E-03 | 1.11[1.03-1.19] | 67.0% | 68.0% | 0.208 | 0.93[0.84-1.04] | 64.9% | 61.9% | 0.768 | 1.02[0.89-1.16] | 0.114 | 1.05 | **0.676** | **1.02** |
| *TLR8* intron1 | I | rs5744055 | 12932833 | G | 15.9% | 16.0% | 0.773 | 0.99[0.89-1.09] | -- | -- | -- | -- | 26.4% | 28.9% | 2.7E-03 | 0.80[0.69-0.93] | -- | -- | -- | -- |
| *TLR8* intron1 | I | rs5744057 | 12933360 | T | 15.9% | 16.0% | 0.770 | 0.99[0.89-1.09] | -- | -- | -- | -- | 26.4% | 28.9% | 2.7E-03 | 0.80[0.69-0.93] | -- | -- | -- | -- |
| *TLR8* intron1 | I | rs5744061 | 12933706 | T | -- | -- | -- | -- | 3.7% | 4.2% | 0.249 | 0.86[0.67-1.11] | -- | -- | -- | -- | -- | -- | -- | -- |
| *TLR8* intron1 | I | rs5744062 | 12933819 | G | -- | -- | -- | -- | 3.7% | 4.2% | 0.245 | 0.86[0.67-1.11] | -- | -- | -- | -- | -- | -- | -- | -- |
| *TLR8* intron1 | I | rs193131573 | 12933848 | C | -- | -- | -- | -- | 2.0% | 1.4% | 0.099 | 1.39[0.94-2.07] | -- | -- | -- | -- | -- | -- | -- | -- |
| *TLR8* intron1 | I | rs5741886 | 12934294 | A | 41.5% | 39.2% | 7.9E-03 | 1.10[1.03-1.19] | -- | -- | -- | -- | 65.7% | 62.8% | 0.842 | 1.01[0.89-1.16] | -- | -- | -- | -- |
| *TLR8* intron1 | I | rs5744067 | 12934973 | C | 19.4% | 17.2% | 1.1E-03 | 1.17[1.06-1.28] | -- | -- | -- | -- | 32.3% | 27.8% | 0.018 | 1.18[1.03-1.36] | -- | -- | -- | -- |
| *TLR8* intron1 | I | rs5744068 | 12935058 | T | 15.8% | 16.0% | 0.658 | 0.98[0.89-1.08] | -- | -- | -- | -- | 25.5% | 27.4% | 7.1E-03 | 0.82[0.71-0.95] | -- | -- | -- | -- |
| *TLR8* intron1 | I | rs5744074 | 12936403 | C | -- | -- | -- | -- | 10.3% | 10.1% | 0.646 | 1.04[0.88-1.23] | -- | -- | -- | -- | -- | -- | -- | -- |
| *TLR8* intron1 | I | rs191344038 | 12936486 | C | -- | -- | -- | -- | 2.0% | 1.4% | 0.093 | 1.41[0.94-2.09] | -- | -- | -- | -- | -- | -- | -- | -- |
| *TLR8* exon2 | G | rs2159377 | 12937513 | T | 19.4% | 17.2% | 1.1E-03 | 1.17[1.06-1.28] | 15.7% | 14.6% | 0.170 | 1.10[0.96-1.27] | 32.6% | 28.1% | 0.017 | 1.18[1.03-1.36] | **2.5E-05** | **1.16** | 2.5E-05 | 1.16 |
| *TLR8* exon2 | G | rs5744080 | 12937804 | T | 41.0% | 38.8% | 9.2E-03 | 1.10[1.02-1.18] | 77.3% | 77.1% | 0.979 | 1.00[0.89-1.13] | 65.0% | 61.7% | 0.639 | 1.03[0.90-1.18] | **0.025** | **1.07** | 0.025 | 1.07 |
| *TLR8* exon2 | G | rs2407992 | 12939112 | C | 41.4% | 39.1% | 6.5E-03 | 1.11[1.03-1.19] | 89.8% | 89.5% | 0.884 | 1.01[0.86-1.20] | 66.0% | 63.5% | 0.870 | 0.99[0.87-1.13] | **0.027** | **1.07** | 0.128 | 1.06 |
| *TLR8* exon2 | I | rs5744082 | 12939303 | A | -- | -- | -- | -- | 2.0% | 1.4% | 0.093 | 1.41[0.94-2.09] | -- | -- | -- | -- | -- | -- | -- | -- |
| *TLR8* exon2 | G | rs3747414 | 12939412 | A | 35.5% | 33.3% | 6.8E-03 | 1.11[1.03-1.20] | 38.2% | 36.6% | 0.224 | 1.07[0.96-1.18] | 58.7% | 56.6% | 0.628 | 0.97[0.85-1.10] | **0.015** | **1.07** | 0.106 | 1.06 |
| *TLR8* 3'UTR | I | rs5744085 | 12940291 | A | -- | -- | -- | -- | 1.9% | 1.4% | 0.088 | 1.42[0.95-2.12] | -- | -- | -- | -- | -- | -- | -- | -- |
| *TLR8* 3'UTR | I | rs5744086 | 12940375 | T | 5.6% | 5.4% | 0.672 | 1.04[0.88-1.21] | -- | -- | -- | -- | -- | -- | -- | -- | -- | -- | -- | -- |
| *TLR8* 3'UTR | I | rs5744088 | 12940564 | C | 15.8% | 16.0% | 0.735 | 0.98[0.89-1.08] | -- | -- | -- | -- | 25.9% | 28.2% | 2.4E-03 | 0.80[0.69-0.92] | -- | -- | -- | -- |
| *TLR8* 3'UTR | I | rs5744089 | 12940639 | A | -- | -- | -- | -- | 1.9% | 1.4% | 0.088 | 1.42[0.95-2.12] | -- | -- | -- | -- | -- | -- | -- | -- |
| *TLR8* 3' downstream | I | rs62590554 | 12941538 | G | 16.2% | 16.3% | 0.868 | 0.99[0.90-1.09] | -- | -- | -- | -- | 25.6% | 27.6% | 4.8E-03 | 0.81[0.70-0.94] | -- | -- | -- | -- |
| *TLR8* 3' downstream | I | rs16987224 | 12942210 | G | 15.8% | 16.0% | 0.803 | 0.99[0.90-1.09] | -- | -- | -- | -- | -- | -- | -- | -- | -- | -- | -- | -- |
| *TLR8* 3' downstream | I | rs112664771 | 12943671 | T | 5.6% | 5.4% | 0.691 | 1.03[0.88-1.21] | -- | -- | -- | -- | -- | -- | -- | -- | -- | -- | -- | -- |
| *TLR8* 3' downstream | I | rs149150348 | 12947309 | C | 5.6% | 5.4% | 0.695 | 1.03[0.88-1.21] | -- | -- | -- | -- | -- | -- | -- | -- | -- | -- | -- | -- |
| *TLR8* 3' downstream | I | rs9781405 | 12947314 | G | 5.6% | 5.4% | 0.695 | 1.03[0.88-1.21] | -- | -- | -- | -- | -- | -- | -- | -- | -- | -- | -- | -- |
| *TLR8* 3' downstream | I | rs73449637 | 12949357 | A | 5.6% | 5.4% | 0.671 | 1.04[0.88-1.21] | -- | -- | -- | -- | -- | -- | -- | -- | -- | -- | -- | -- |
| Abbreviation: G, genotype; I, imputed; OR, odds ratio; --, missing data | | | | | | | | | | | | | | | | | | |  |  |
| Position of each SNP is based on GRch37/hg19. | | | | | | | | | | | | | | | | | | |  |  |
| Seven SNPs that showed consistent association with SLE (*P*<0.05) in all 3 ancestral groups are highlighted in gray. | | | | | | | | | | | | | | | | | | |  |  |
| The meta-analysis was performed with both fixed and random-effects model. If the Cochran's Q statistic showed no evidence of genetic heterogeneity (*P* >0.05), | | | | | | | | | | | | | | | | |  |  |  |  |
| the *P* value and OR from a fixed effect model was applied. Otherwise, a random effect model was used. The finally applied meta *P* value and OR for each SNP are highlighted in bold. | | | | | | | | | | | | | | | | | | |  |  |
